# Precursor-Dependent Performance of FA-, GBFS-, MK- and WBP-Based Geopolymer Mortars: Effects of NaOH Molarity and Thermal Curing on Strength, Transport Properties and Cost Efficiency

**DOI:** 10.3390/polym18141723

**Published:** 2026-07-13

**Authors:** Damla Nur Çelik, Rüya Kılıç Demircan, Güneş Mutlu Avinç, Gökhan Kaplan

**Affiliations:** 1 Department of Civil Engineering, Faculty of Technology, Gazi University, Ankara 06500, Türkiye; damlanur.celik@gazi.edu.tr; 2 Department of Construction Technology, Boyabat Vocational School, Sinop University, Sinop 57200, Türkiye; 3 Department of Architecture, Faculty of Architecture and Fine Arts, Ankara Yıldırım Beyazıt University, Ankara 06760, Türkiye; gunesavinc@aybu.edu.tr; 4 Department of Civil Engineering, Engineering Faculty, Atatürk University, Erzurum 25240, Turkey

**Keywords:** alkali activation, chloride-ion penetration, cost index, electrical resistivity, waste brick powder

## Abstract

This study investigated the effects of precursor type, NaOH molarity, and thermal curing temperature on the performance of geopolymer mortars produced using fly ash (FA), ground granulated blast-furnace slag (GBFS), metakaolin (MK), and waste brick powder (WBP). Mortars were activated using 12 M and 16 M NaOH solutions at a constant Na_2_SiO_3_/NaOH ratio and thermally cured at 60 and 90 °C for 24 h. Physical, mechanical, transport, microstructural, and cost-performance properties were evaluated. The results demonstrated that the optimum activation conditions strongly depended on precursor type. MK-based mortars cured at 16 M–90 °C exhibited the best overall performance, achieving the lowest apparent porosity (6.1%) and water absorption (5.4%), and the highest oven-dry density (2194 kg/m^3^), compressive strength (25.8 MPa), and flexural strength (3.43 MPa). These mortars also exhibited the lowest capillary water absorption (1.88 kg/m^2^), the highest electrical resistivity (248.00 kΩ·cm), and the lowest charge passed (177 C), indicating enhanced pore refinement and chloride-ion penetrability. In contrast, GBFS performed better under milder activation conditions, whereas WBP showed lower performance due to its coarser, more crystalline structure. SEM/EDS analyses confirmed that the formation of dense aluminosilicate gel governed matrix quality and overall performance. Overall, MK activated at 16 M and cured at 90 °C provided the most favorable balance between technical performance and cost efficiency.

## 1. Introduction

The construction sector faces mounting pressure to reduce its environmental impact while meeting the growing demand for building materials. Buildings and construction activities account for a significant portion of global energy consumption and CO_2_ emissions. Additionally, the extraction, processing, transportation, and disposal of construction materials exacerbate resource depletion and waste production [1,2]. This challenge has shifted the focus from solely enhancing the mechanical properties of cement-based materials to developing binders that are reliable, resource-efficient, and aligned with circular-economy principles. In this regard, geopolymer and alkali-activated materials have gained interest as viable alternatives because they can convert aluminosilicate-rich industrial by-products and mineral wastes into valuable construction materials [3,4]. Geopolymers were first developed as synthetic aluminosilicate binders created through the reaction of a reactive Si–Al source with an alkaline activating solution [5]. Unlike traditional Portland cement, which requires high-temperature clinkerization and emits CO_2_ during production, geopolymers can be made from low-calcium or calcium-rich aluminosilicate materials such as fly ash, blast furnace slag, metakaolin, ceramic waste, brick powder, and other industrial byproducts [6,7]. Their bonding mechanism involves dissolving Si and Al species under alkaline conditions, followed by polycondensation, resulting in three-dimensional aluminosilicate networks or calcium-modified reaction products [8,9,10]. Thus, the properties of geopolymer composites are influenced by various factors, including precursor characteristics, the amount of amorphous phase, particle size, activator type, and curing process, rather than by a single reaction pathway.

Fly ash (FA), ground granulated blast furnace slag (GBFS), metakaolin (MK), and waste brick powder (WBP) are four distinct precursor types with different reaction behaviors. Low-calcium Class F fly ash mainly forms N-A-S-H-type gels but reacts slowly at ambient conditions or with insufficient heat due to slow dissolution kinetics [11,12]. Conversely, GBFS contains high CaO levels and reactive glassy phases that facilitate the quick formation of C-S-H/C-A-S-H/C-(N)-A-S-H-type products, leading to rapid early-age strength gain [13,14]. MK is a highly reactive, dehydroxylated aluminosilicate rich in Al_2_O_3_ and SiO_2_; however, its high surface area and rapid dissolution make its performance highly sensitive to alkalinity and curing temperature [15,16,17]. WBP is appealing for waste valorization because it is rich in aluminosilicates from construction and demolition waste. WBP was incorporated as a low-cost construction waste, characterized by high SiO_2_–Al_2_O_3_ content but limited intrinsic reactivity due to its crystalline fired-clay structure. This made it an appropriate precursor for studying how a less reactive, waste-derived aluminosilicate responds to the same NaOH molarity and thermal curing conditions used for FA, GBFS, and MK. Therefore, WBP enabled a direct assessment of the balance among waste valorization, technical performance, and cost efficiency. Still, it’s more crystalline structure, coarser particles, and the presence of low-reactivity quartz/mullite phases can restrict geopolymerization unless enhanced by sufficient activation or blending strategies [18,19]. The alkaline activator system is essential for transforming precursors into solid geopolymer matrices. Sodium hydroxide raises the pH and accelerates the dissolution of aluminosilicate materials, while sodium silicate provides soluble silica that aids gel formation and densifies the matrix [20,21,22]. However, simply increasing NaOH molarity does not always improve performance. Low molarity may not effectively dissolve Si and Al, and too high alkalinity can reduce workability, accelerate setting times, raise free alkali levels, and produce uneven reaction products or microcracks [23]. Similarly, thermal curing can boost early geopolymerization, especially in low-calcium fly ash and WBP systems. Still, excessive thermal treatment may cause water loss, shrinkage microcracks, and pore coarsening in calcium-rich systems [24,25,26]. Consequently, the optimal molarity and curing temperature are likely dependent on the precursor type rather than being universally applicable.

In addition to compressive strength, the suitability of geopolymer composites for engineering applications relies on their pore structure and resistance to ion transport. Key indicators such as apparent porosity, water absorption, and capillary water absorption reveal details of the open, interconnected pore network. Meanwhile, electrical resistivity and ASTM C1202 [27] charge passed are common methods for evaluating ionic transport and chloride-ion penetration. However, interpreting transport tests in geopolymer systems requires caution, as the electrical response depends not only on pore connectivity but also on the chemistry of the pore solution, especially the presence of mobile Na^+^, K^+^ and OH^−^ ions [28]. Studies indicate that chloride penetration and steel corrosion resistance in fly ash-based geopolymers are influenced by NaOH concentration and matrix compactness [29], while the electrical resistivity of FA- and MK-based geopolymers varies with precursor type, activator conditions, and curing regime [30,31,32,33]. Therefore, a comprehensive durability assessment should integrate physical pore indicators, mechanical performance, electrical resistivity, and chloride-ion penetration rather than evaluating each parameter in isolation.

While much research has explored individual geopolymer precursors, several gaps persist. Many studies concentrate on a single binder type, limited activator conditions, or focus solely on mechanical performance. Others evaluate transport properties or environmental impacts separately, without connecting them to precursor characteristics or cost efficiency. Recent scientometric and review studies reveal that geopolymers are gaining attention in green building and circular economy research, yet key challenges remain in material formulation, performance optimization, durability, and feasibility [4]. Additionally, research on WBP-based geopolymers highlights brick waste as a promising circular precursor. Still, it stresses the need for a better understanding of its fresh and hardened properties, reaction kinetics, and microstructural constraints [34,35]. From an engineering standpoint, direct comparisons among FA, GBFS, MK, and WBP using the same activator ratios, molarity levels, and thermal curing conditions are scarce, especially when evaluating physical, mechanical, transport, and cost-performance factors collectively.

Beyond their engineering performance, geopolymer-based materials also present significant opportunities for architectural applications due to their lower environmental impact, material versatility, and compatibility with circular construction approaches. The use of industrial by-products and construction and demolition waste as precursors supports sustainable architectural design strategies by reducing embodied carbon, minimizing landfill disposal, and promoting resource-efficient material cycles. In addition, the ability to tailor pore structure, durability, and surface characteristics through activator chemistry and curing conditions makes geopolymer mortars promising candidates for façade panels, masonry units, repair mortars, paving elements, and prefabricated architectural components. Therefore, understanding the relationship between precursor characteristics and performance is important not only for structural engineering but also for the development of sustainable and resilient architectural materials.

This study presents a systematic, comparative evaluation of geopolymer mortars using four aluminosilicate binders—FA, GBFS, MK, and WBP—tested at two NaOH molarities (12 M and 16 M) and two curing temperatures (60 °C and 90 °C). The mixture design kept binder content and the Na_2_SiO_3_/NaOH ratio constant to isolate the effects of precursor type, alkalinity, and curing temperature. The research assesses parameters including oven-dry density, apparent porosity, water absorption, compressive and flexural strength, capillary water absorption, electrical resistivity, and chloride-ion penetration. Additionally, a simplified cost analysis and index are included to link technical performance to economic efficiency. The significance of this study lies in establishing a direct link among precursor characteristics, activation conditions, microstructural development, transport resistance, and cost-normalized performance. Rather than merely identifying the best-performing mixture, the study clarifies why each precursor responds differently to the same molarity and curing regimes. Therefore, the findings provide a practical, performance-based framework for selecting and optimizing geopolymer binders for durable, resource-efficient, and cost-effective construction applications.

## 2. Materials and Methods

### 2.1. Raw Materials

In this study, four aluminosilicate-based binders were used to synthesize geopolymer composites: Fly Ash (FA), Granulated Blast Furnace Slag (GBFS), Metakaolin (MK), and Waste Brick Powder (WBP). FA was sourced from the Çatalağzı Thermal Power Station (Zonguldak, Türkiye), GBFS from Oyak Cement (Zonguldak, Türkiye), and WBP from a local brick production plant (Sinop, Türkiye). MK was sourced from a Chinese manufacturer via a local supplier.

FA has a specific gravity of 2.23 and falls under ASTM C618 Class F [36]. Chemical analysis shows that SiO_2_ + Al_2_O_3_ + Fe_2_O_3_ totals 90.6%, while calcium oxide (CaO) is only 4.3%, indicating it is a suitable precursor for forming a stable aluminosilicate network. GBFS has a specific gravity of 2.87 and complies with ASTM C989 standards [37], containing 42.3% CaO and serving as the primary calcium source. It also contains 10.8% MgO. MK, a highly reactive auxiliary binder, has a specific gravity of 2.44 and mainly consists of Al_2_O_3_ (43.4%) and SiO_2_ (51.9%). It is dehydroxylated; its mostly amorphous structure makes it suitable for geopolymerization. WBP was produced by recycling brick fragments from a brick factory. The fragments were first crushed to 0–1 mm, then ground in a ball mill for 90 min to achieve the desired fineness. WBP has a specific gravity of 2.42, with 48.9% SiO_2_ and 24.2% Al_2_O_3_. Its low LOI of 0.6% suggests it is a well-calcined precursor. The chemical compositions of these binding materials are presented in Table 1.

**Figure 1:**
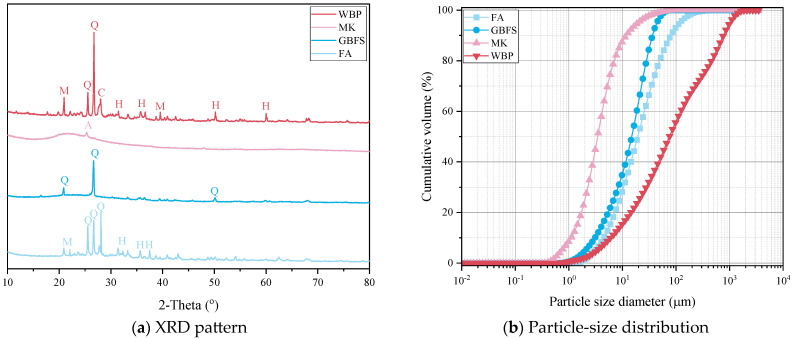
The mineralogical and physical properties of binders.

The mineralogical composition and particle size distribution of the binding agents are presented in Figure 1. Examination of the XRD patterns in Figure 1a shows a broad amorphous peak (hump) between 20° and 30° (2θ) in the FA and MK samples, indicating a high proportion of reactive glass phase. In the MK sample, the XRD pattern showed a broad amorphous hump between approximately 20° and 30° (2θ), indicating a predominantly amorphous aluminosilicate nature. Besides this amorphous region, a faint reflection attributed to anatase (TiO_2_) appeared as a minor crystalline impurity, likely originating from Ti-rich accessory minerals in the parent kaolin. Consequently, anatase was not regarded as a significant component of MK but rather as a trace/minor phase observed in the XRD pattern. Mullite (M) and Quartz (Q) crystals are present in the FA structure. GBFS exhibited a predominantly amorphous/glassy structure, whereas WBP, unlike the other binders, presents a more crystalline matrix; the phases predominantly identified in its structure are Calcite (C), Quartz (Q), Hematite (H), and Mullite (M). In particular, the presence of Hematite (H) in WBP is consistent with the high Fe_2_O_3_ content in chemical analyses and the material’s characteristic color. The particle size distribution (PSD) curves in Figure 1b provide essential insights into the material’s fineness and packing potential. MK showed the finest distribution, with about 90% of its particles (d_90_) smaller than 10 µm, likely leading to high reactivity and strong early-age strength. Although GBFS and FA present similar gradation profiles, the d_50_ of GBFS was around 15 µm. After 90 min of grinding, WBP showed a coarser distribution than the other precursors, with a d_50_ of about 60 µm. Its broader particle-size distribution suggests it may serve as a filler in the composite, thereby enhancing the matrix density. Nonetheless, its larger average particle size could negatively impact geopolymerisation.

**Figure 2:**
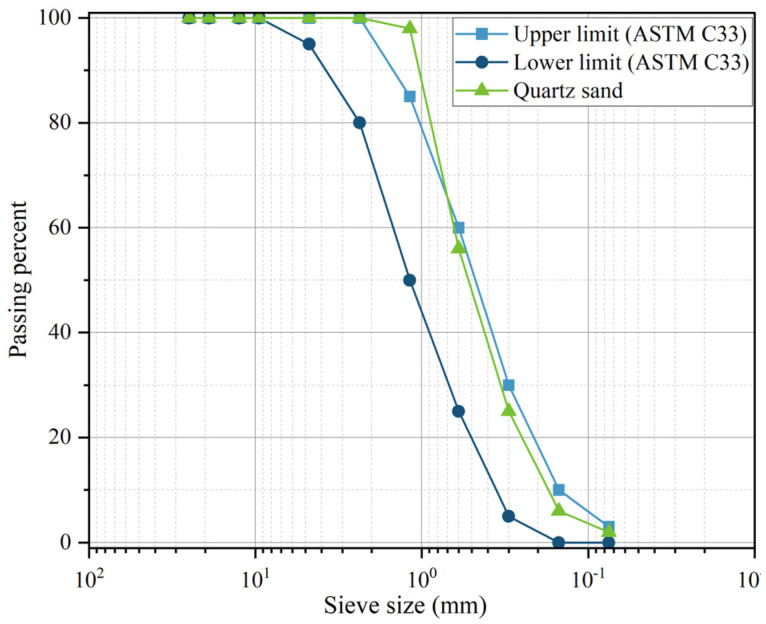
Size distribution of quartz sand based on sieve analysis.

The study used quartz sand as the fine aggregate, characterized by a high silica content and particle sizes ranging from 0 to 1 mm. The quartz sand was sourced from Aydınlar Madencilik (İzmir, Türkiye). Its specific gravity was measured at 2.77, with a water absorption capacity of 0.92%. The aggregate’s particle size distribution was compared to the ASTM C33 standard [38] limits, based on sieve analysis results shown in Figure 2. The gradation curve of the quartz sand falls within the standard’s specified lower and upper limits. Notably, the particle density in the 0.15–0.6 mm range promotes a homogeneous distribution in the geopolymer matrix and helps form a compact structure. Additionally, the aggregate’s low water absorption helped maintain the workability of fresh geopolymer mixtures and enabled precise control of the water-to-solid ratio in the activator solution.

In this study, a binary alkali activator system consisting of sodium silicate (Na_2_SiO_3_) and sodium hydroxide (NaOH) solutions was used to activate aluminosilicate precursors. The alkaline components, consisting of NaOH and Na_2_SiO_3_, were sourced from Sigma-Aldrich. The NaOH solutions were prepared to the final solution volume. For the 12 M NaOH solution, 480 g of NaOH pellets was slowly added to approximately 600–700 mL of distilled water under continuous stirring. After complete dissolution, the solution was allowed to cool to room temperature because the dissolution of NaOH is exothermic. The cooled solution was then transferred to a 1 L volumetric flask, and distilled water was added until the final volume reached exactly 1 L. The same procedure was followed for the 16 M NaOH solution, dissolving 640 g of NaOH pellets and diluting the cooled solution to a final volume of 1 L. The prepared NaOH solutions were stored in sealed containers and kept at laboratory temperature for 24 h before mixing. Therefore, the reported 12 M and 16 M values correspond to the nominal molarity of the NaOH stock solutions prior to incorporation into the geopolymer mixtures. The liquid sodium silicate (commonly known as water glass) used in the study was obtained commercially. The silica modulus (Ms = SiO_2_/Na_2_O) of the Na_2_SiO_3_ solution is 2. Incorporating sodium silicate into the system not only increases alkalinity but also enhances geopolymerization rate and matrix density by providing readily soluble silica to the environment.

### 2.2. Mix Design

In this study, 12M and 16M denote the nominal molarity of the pre-made NaOH stock solutions before their addition to the mortar mixtures. The extra water listed in Table 2 was not used to prepare the NaOH stock but was added separately during mixing to control workability and slow the reaction, especially in the 16 M mixes. Once all liquid components were combined in the fresh paste, this extra water became part of the total liquid phase and inevitably lowered the effective hydroxide concentration of the overall activator system. As a result, the molarity values reported here should be viewed as the initial NaOH solution molarity, not the final diluted molarity of the entire pore solution. During mortar production, the quantity of quartz sand was adjusted to achieve the target total mixture volume, which ranged from approximately 883 to 1092 kg/m^3^.

The mixtures were prepared with a constant binder mass of 600 kg/m^3^ for all systems, enabling a comparison of different aluminosilicate precursors under identical conditions such as binder and activator dosages, Na_2_SiO_3_/NaOH ratio, and curing parameters. Because the precursors have different specific gravities, their actual binder volumes varied: approximately 0.269 m^3^/m^3^ for FA, 0.209 m^3^/m^3^ for GBFS, 0.246 m^3^/m^3^ for MK, and 0.248 m^3^/m^3^ for WBP. To keep the total mixture volume consistent, the quartz sand content was adjusted for each mix. Consequently, the comparison primarily reflects performance at equal binder mass, not volume. The specific gravity of the precursors also influenced the interpretation of oven-dry density and porosity results.

The comparison between 12 M and 16 M mixtures considers the combined effects of nominal NaOH molarity and the adjusted extra water content (Table 3). While the activator-to-binder ratio based on NaOH and Na_2_SiO_3_ solutions remained constant at 0.70, the total liquid-to-binder ratio rose from 0.783 to 0.817 as the NaOH molarity increased from 12 M to 16 M. This extra water was added to offset the reduced workability and faster reaction rates of the 16 M mixes.

To clarify how additional water influences the effective alkalinity of our mixtures, we distinguished between the nominal NaOH stock molarity and the adjusted NaOH concentration after water was added (Table 4). The values of 12 M and 16 M denote the initial molarities of the NaOH solutions before mixing. Since we added 50 kg/m^3^ and 70 kg/m^3^ of extra water to the 12 M and 16 M mixtures, respectively, we recalculated the effective NaOH concentrations based on the volumes of NaOH stock solution and water added. Using measured densities of 1.38 kg/L for 12 M and 1.43 kg/L for 16 M, the adjusted NaOH concentrations were found to be 7.62 M and 8.72 M, respectively. Consequently, comparing the 12 M and 16 M series reflects not only the nominal NaOH molarity but also the effects of dilution, additional water, and total liquid-to-binder ratio, rather than NaOH concentration effects alone.

### 2.3. Mixing, Casting and Curing

The mixing process began with preparing the paste. NaOH, Na_2_SiO_3_, and excess water were sequentially added to the binder; the mixture was stirred for 1 min at low speed, then for 1 min at high speed. In the second step, quartz sand was added to the mixture, which was stirred for 1 min at low speed and 3 min at high speed to produce fresh geopolymer mixtures. A laboratory-grade Hobart mixer was used to prepare the mixtures. After the fresh geopolymer mixtures were placed in their molds, their surfaces were covered with stretch film. Immediately afterward, the molds were placed in an oven, initiating the heat-curing process. An oven with a heating rate of 5 °C/min was used for the heat curing. The specimens were cured at 60 and 90 °C for 24 h and allowed to cool slowly in the oven. The specimens were then removed from their molds and left to stand under laboratory conditions (23 ± 2 °C and 40 ± 5% RH) until the day of testing. For each mixture and test age, at least three replicate specimens were tested, and the average values were reported.

### 2.4. Test Methods

Tests were conducted to determine the physical characteristics of geopolymer composites, including oven-dry density, apparent porosity, and water absorption capacity. For the physical-property tests, cubic specimens measuring 50 × 50 × 50 mm from each series were evaluated at 28 days, the standard age for hardened-state characterization. Tests for density, water absorption, and apparent porosity were conducted in accordance with ASTM C642 [39]. The 28-day benchmark was selected to maintain consistency with the assessments of mechanical and transport properties, facilitating comparisons across different precursor systems. This age does not indicate the completion or full stabilization of the geopolymerization process, which can continue beyond 28 days depending on the precursor characteristics and curing conditions. To remove initial free water and ensure a constant mass, the specimens were dried in an oven at 50 °C for 3 days. A low drying temperature (50 °C) was chosen to prevent microcracks and structural damage in the geopolymer matrix.

Flexural and compressive strength tests were conducted to evaluate the mechanical performance of geopolymer mortars. During the experiments, days 1, 7, and 28 were selected as test ages to monitor the time-dependent development of geopolymerization. Flexural strength tests were performed on prismatic specimens measuring 40 × 40 × 160 mm, taken from each series, in accordance with ASTM C348 [40]. The tests were conducted on a three-point bending rig with a single load applied to the center of the specimens. Specimens (half-prisms) that were split into two pieces as a result of the bending test were used for the compression test to ensure continuity of the mechanical properties. These tests were carried out in accordance with ASTM C349 [41]. The load was applied to the side surfaces of the specimens (perpendicular to the casting direction) using standard 40 × 40 mm steel plates. For each test age, the final strength values were reported as the average of three prism specimens.

The capillary water absorption test was adapted from TS EN 1015-18 [42] principles, using 50 × 50 × 50 mm cube specimens. This size was chosen to ensure consistency with other physical-property tests and to allow direct comparison of density, water absorption, apparent porosity, and capillary uptake results, all using the same specimen geometry. Additionally, the 50 mm cube provided enough exposed surface area for unidirectional water absorption while enabling uniform sealing of the sides with waterproofing material. Since the main goal was to compare the capillary absorption behavior of different precursor-based geopolymers rather than establish a formal standard, the results were treated as comparative transport indicators. Thus, 50 mm cube specimens were deemed appropriate for this study.

Electrical resistivity measurements were carried out on prismatic specimens measuring 40 × 40 × 160 mm that had completed a 28-day curing period. In accordance with the ASTM C1876 standard [43], the ‘Bulk Resistivity’ method was employed for the measurements. During testing, care was taken to ensure the specimens were in a saturated surface-dry (SSD) condition; the electrical resistance between two electrodes was measured, and the bulk resistivity (kΩ·cm) was calculated from the specimen geometry.

**Figure 3:**
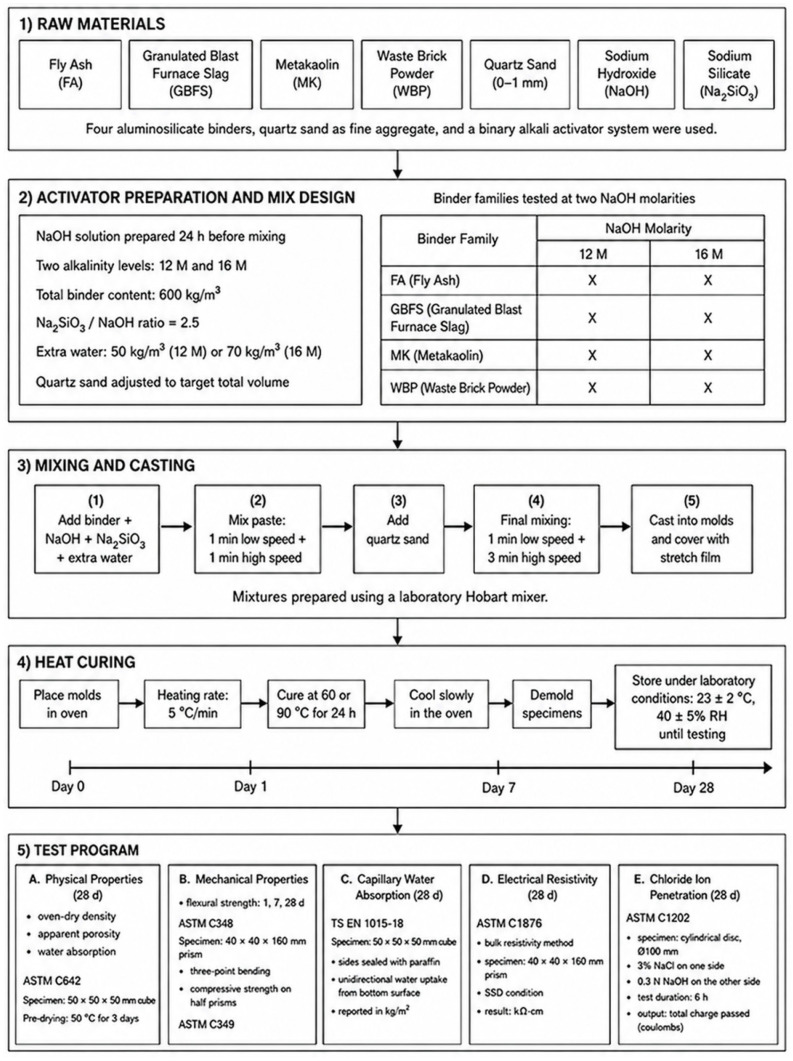
Flowchart of the experimental study.

The ASTM C1202 standard [27] was used to assess chloride-ion penetration resistance. For this test, cylindrical disc specimens with a diameter of 100 mm and a thickness of 50 mm were used. During the test, one side of each specimen was exposed to a 3% NaCl solution, while the opposite side faced a 0.3 N NaOH solution. The total electrical charge (in Coulombs) passing through each specimen over six hours was measured. This charge was then used to classify the chloride-ion penetrability of the samples as very low, low, medium, or high. The flowchart for the experimental study is shown in Figure 3.

SEM/EDS analyses were conducted to investigate the microstructural features and local chemical composition of specific geopolymer mixtures. Samples were taken from the inner fractured surfaces of 28-day specimens post-mechanical testing, avoiding the external casting surface. The fragments were carefully cleaned to remove loose particles and dried at 50 °C for 24 h to eliminate free moisture without causing additional thermal damage. The dried samples were mounted on aluminum stubs using conductive carbon tape. Because geopolymer matrices are non-conductive, they were coated with a thin layer of conductive gold (Au) before SEM/EDS analysis. SEM imaging was performed at various magnifications to identify unreacted or partially reacted precursor particles, gel regions, open pores, microcracks, and secondary reaction products. EDS point analysis was performed to determine the local elemental compositions of the regions observed in the SEM images. The EDS results were interpreted alongside the SEM morphology, basic chemistry, and expected reaction products of the alkali-activated systems. Accordingly, the Na–Al–Si-rich region was assigned to N-A-S-H-type gel reaction products, while the Ca–Si–Al-rich region was assigned to C-A-S-H/C-(N)-A-S-H-type reaction products. These assignments were considered SEM/EDS-supported microstructural interpretations rather than direct quantitative phase determinations.

## 3. Results and Discussion

### 3.1. Physical Properties

It is important to remember that differences in particle size distribution among precursor materials are key to their properties and can affect their behaviour in the fresh state, bulk density, reaction kinetics, porosity development, and strength. Consequently, the effects seen in this study should not be attributed only to chemical composition or NaOH molarity. Instead, the performance of the precursor materials is understood as a combined result of chemical composition, mineralogical structure, amorphous phase content, particle size distribution, and curing or activation conditions.

**Figure 4:**
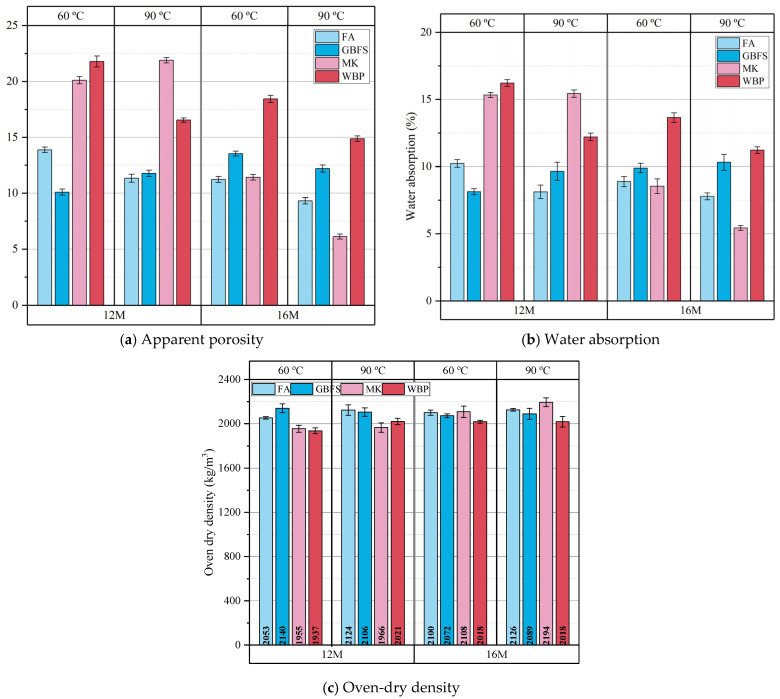
The physical properties of geopolymer samples.

Generally, there is a direct correlation between apparent porosity and water absorption, whilst there is usually an inverse relationship between oven-dry density and porosity (Figure 4). Oven-dry density should not be viewed only as an indicator of pore volume. In multi-precursor geopolymer mortars, density depends on both the matrix’s compactness and the solid parts’ specific gravity. Hence, a higher oven-dry density does not automatically mean the mixture has the lowest apparent porosity. This is especially true for GBFS-based mixes, as GBFS has a higher specific gravity than FA, MK, and WBP; thus, GBFS mixtures can appear relatively dense even if their apparent porosity is not the lowest. The chemical/mineralogical differences of the FA, GBFS, MK and WBP used in the study, together with the curing conditions of 12–16 M NaOH and 60–90 °C, are the main variables directly controlling these physical results.

Higher NaOH molarity and curing temperatures lead to a denser structure in FA-based mixes. Under 12 M–60 °C, FA samples showed an apparent porosity of 13.9%, which decreased to 9.3% at 16 M–90 °C. Likewise, water absorption dropped from 10.2% to 7.8%, and oven-dry density rose from 2053 kg/m^3^ to 2126 kg/m^3^. This trend occurs because, in low-calcium Class F fly ash systems, increased alkalinity and heat treatment enhance the dissolution of Si and Al, promoting N-A-S-H gel formation [44]. It is known that proper thermal curing in low-calcium FA geopolymers reduces permeable voids and sorptivity, resulting in a denser, more durable matrix [44]. Thus, the lower porosity and water absorption at 16 M–90 °C are due to greater activation and fewer interconnected capillary voids.

GBFS-based mixtures showed varying behaviors. The lowest apparent porosity and water absorption were observed at 12 M–60 °C, with porosity at 10.1% and water absorption at 8.1%. Conversely, at 16 M, porosity increased to 13.5% and 12.2%, and water absorption rose to 9.9% and 10.3%. This suggests that GBFS, with its high CaO content, is already highly reactive. A higher molarity or temperature does not always lead to more densification. In slag systems, a calcium-rich environment can speed up the formation of C-A-S-H/C-(N)-A-S-H gels, creating a dense matrix early on. However, excessive alkalinity or high temperatures can adversely affect pore structure, leading to rapid reactions, water loss, microcracks due to shrinkage, and more heterogeneous gel formation [45,46]. Optimal physical performance at 12 M–60 °C indicates that this binder reacts well under moderate activation, whereas more aggressive conditions might partially disrupt microstructural continuity.

The most notable change in MK-based mixtures occurred when NaOH molarity increased from 12 M to 16 M. Under 12 M conditions, the MK samples exhibited very high porosity and water absorption; apparent porosity was measured at 20.1% at 60 °C and 21.9% at 90 °C. Water absorption also remained at approximately 15.3–15.4%. In contrast, at 16 M–60 °C, porosity fell to 11.4%, and at 16 M–90 °C, it dropped to just 6.1%. Water absorption similarly decreased from 8.5% to 5.4%. The oven-dry density reached 2194 kg/m^3^ at 16 M–90 °C—the highest value among all mixtures—indicating that the 16 M–90 °C combination in the MK system forms a much denser network of reaction products. Although MK’s very fine particle size and high Al_2_O_3_ content make it a highly reactive precursor, sufficient alkalinity and appropriate thermal activation are required to fully exploit its reactivity. The literature reports that curing temperature significantly affects the development of the hardened structure and alters the rate of formation of reaction products in MK-based geopolymers [47,48]. The results of this study also demonstrate that 12 M activation is insufficient for MK, whereas the 16 M–90 °C condition significantly increases aluminosilicate dissolution and the continuity of the gel network.

WBP-based mixtures generally retained the highest porosity and water absorption. In the WBP samples, apparent porosity was 21.8% at 12 M–60 °C—one of the highest values across all series—whilst water absorption reached 16.2%. Curing at 90 °C and activation at 16 M partially reduced the porosity; the best WBP result was obtained at 16 M–90 °C with 14.9% porosity and 11.2% water absorption. However, these values remain higher than those under the optimum conditions for MK and FA. This behavior can be attributed to WBP’s coarser particle size, more crystalline mineralogical structure, and the presence of low-reactive phases such as quartz and mullite. The literature highlights that smaller particle sizes in waste clay brick dust can improve the pore structure by enhancing the filler effect and the pozzolanic/alkali activation potential; conversely, coarse, more crystalline WBP may form a more interconnected pore structure [18,49]. Therefore, the partial improvement of WBP under 90 °C and 16 M conditions can be explained by an increase in the degree of activation; however, it is understood that, due to the material’s crystalline nature and low solubility, it cannot form a structure as dense as MK.

The oven-dry density results generally align with the porosity and water absorption data. For the MK mixture, increasing the molarity from 12 M to 16 M increased the density from 1955–1966 kg/m^3^ to 2108–2194 kg/m^3^. This increase is consistent with pores filling with reaction products and the matrix densifying. In FA mixtures, density also increased steadily with rising temperature and molarity. In contrast, although density remained high in GBFS mixtures, the porosity/water absorption values were not always the lowest, indicating that density depends not only on matrix compactness but also on GBFS’s high specific gravity. In WBP mixtures, density values remained within approximately 1937–2021 kg/m^3^, and the relatively high porosity/water absorption values in this binder group suggest that the matrix contains more open or interconnected voids.

Overall, these results indicate that optimal activation conditions vary by binder with respect to physical performance. For FA, 16 M–90 °C proved to be the best condition, resulting in low porosity and water absorption. GBFS performed better at 12 M–60 °C, showing a more balanced, denser structure. MK achieved the highest density and the lowest porosity and water absorption at 16 M–90 °C. Although this temperature improved WBP, the tendency for higher porosity remained in this binder due to its crystalline structure and coarse grains. Ultimately, physical properties depend on the chemical reactivity and mineralogical makeup of the binders: a high amorphous phase and proper activation promote denser gel formation. At the same time, crystalline and coarse structures limit reactions and increase interconnected porosity.

### 3.2. Mechanical Properties

**Figure 5:**
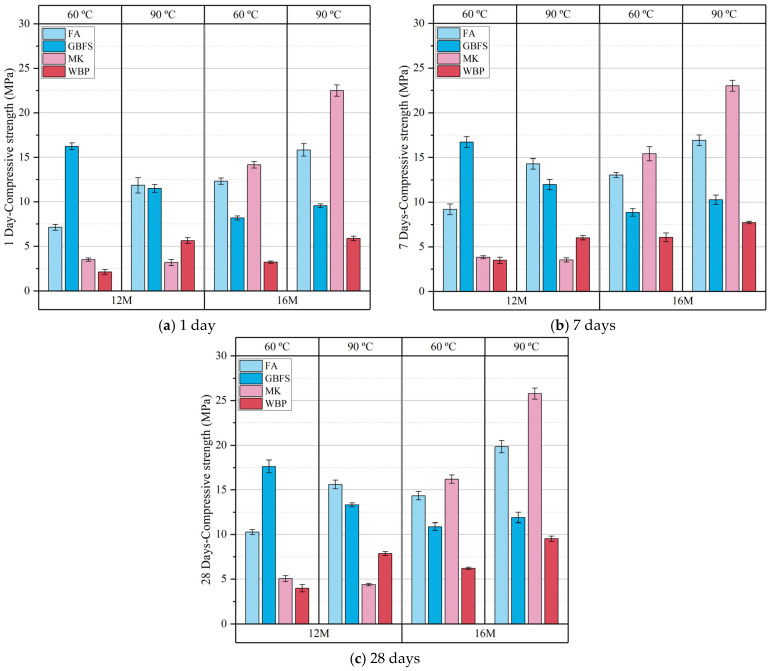
The compressive strength of geopolymer samples.

The 1-, 7-, and 28-day compressive strength results shown in Figure 5 indicate that binder type, NaOH molarity, and curing temperature have significantly different effects on geopolymerization kinetics. In general, FA- and MK-based mixtures achieved higher strength with 16 M NaOH and 90 °C curing, whereas GBFS-based mixtures performed best under 12 M–60 °C conditions. In WBP-based specimens, strength development remained limited across all conditions; however, curing at 90 °C with 16 M activation partially improved WBP’s performance. These results are largely consistent with the previous physical properties: low porosity and low water absorption generally resulted in higher compressive strength. In particular, the 28-day compressive strength reached 25.8 MPa in the MK–16M–90 °C sample, which is directly related to this mixture having the lowest apparent porosity reported in the previous section. These results are consistent with the relationships between binder chemistry, molarity, and curing temperature reported in the literature [50,51,52].

An analysis of FA-based mixtures shows that both NaOH molarity and curing temperature systematically increase strength development. Under 12 M–60 °C conditions, the FA sample’s compressive strength was 7.1 MPa after 1 day, 9.2 MPa after 7 days, and 10.3 MPa after 28 days. When the curing temperature was increased to 90 °C while maintaining the same molarity, the 28-day strength increased to 15.6 MPa. Similarly, while the 28-day strength was 14.4 MPa at 16 M–60 °C, it reached 19.9 MPa at 16 M–90 °C. This behavior can be explained by the more effective dissolution of low-calcium Class F fly ash at higher temperatures than at room temperature or during low thermal activation. Due to FA’s low CaO content, rapid formation of C-A-S-H-type gel at an early age is not expected; instead, strength development depends on the dissolution of Si and Al species in an alkaline environment and the formation of an N-A-S-H-type aluminosilicate gel network. The literature reports that curing temperature and activator concentration are decisive factors affecting compressive strength in FA-based geopolymers, and that high-temperature curing, in particular, accelerates early-age reactions [25,53]. Therefore, the highest strength obtained in FA specimens under 16 M–90 °C conditions can be attributed to the formation of a denser N-A-S-H gel and a reduction in the associated pore structure.

Unlike mixtures with other binders, GBFS-based mixtures reached their peak strength under the 12 M–60 °C condition. In this series, the strengths at 1, 7, and 28 days were 16.2 MPa, 16.7 MPa, and 17.6 MPa. These values indicate that GBFS reacts rapidly at a very early age and attains most of its strength within the first 24 h. In contrast, the 28-day strength dropped to 13.3 MPa at 12 M–90 °C; under 16 M–60 °C and 16 M–90 °C conditions, it remained at 10.9 MPa and 11.9 MPa, respectively. This result indicates that GBFS is already a highly reactive binder due to its high CaO content; therefore, very high alkalinity or high-temperature conditions do not always increase strength. In slag-based alkali-activated systems, the formation of calcium-containing gels of the C-S-H/C-A-S-H/C-(N)-A-S-H type enhances early strength; however, excessively rapid reactions, water loss, possible moisture-loss-related microcracking, and heterogeneous gel precipitation can limit mechanical performance [13,54]. Therefore, the 12 M–60 °C condition provided optimal activation in GBFS; the 16 M and/or 90 °C conditions, however, likely reduced strength due to rapid reaction and a more open pore structure.

The decrease in GBFS performance under conditions of 16 M and/or 90 °C should be interpreted carefully. Since drying shrinkage was not directly measured in this study, the reduced performance cannot be solely attributed to shrinkage. Nonetheless, the experimental results consistently indicate a less favorable matrix structure under more aggressive activation conditions. Compared to the GBFS–12M–60 °C mixture, samples activated at 16 M or cured at 90 °C exhibited higher apparent porosity and water absorption, lower compressive strength, and increased capillary water absorption. SEM analysis of the GBFS–16M–90 °C mixture revealed a heterogeneous reaction matrix with open pores and crack-like features. These results imply that excessive alkalinity and/or higher curing temperatures may accelerate rapid, uneven reactions, cause moisture-loss-related microcracking, and lead to greater pore connectivity. Consequently, the performance decline is considered a plausible mechanism supported by physical, mechanical, transport, and SEM data, rather than a direct measure of shrinkage.

MK-based mixtures are the most sensitive binder group to NaOH molarity. At 12 M, MK samples showed very low strength, with 28-day strengths of 5.1 MPa at 12 M–60 °C and 4.4 MPa at 12 M–90 °C. Increasing the concentration to 16 M resulted in a significant increase in strength: 16.2 MPa at 16 M–60 °C and 25.8 MPa at 16 M–90 °C. This notable rise indicates MK’s strong reaction potential, owing to its high Al_2_O_3_ content and fine grain size, but it also requires adequate alkalinity and thermal energy for activation. The 12 M activation was inadequate for MK, limiting aluminosilicate dissolution and resulting in low strength. At 16 M–90 °C, MK’s amorphous/dehydroxylated structure dissolved more effectively, accelerating Al-O-Si bond formation and creating a denser aluminosilicate gel network. Rovnaník highlighted that the curing temperature greatly influences the development of the hardened structure in MK-based geopolymers, with the amorphous gel phase being crucial for mechanical properties [48]. Conversely, the literature warns that excessively high temperatures can negatively impact MK systems due to water loss and rapid surface gel formation [55]. However, in this study, the positive effect of 90 °C is attributed to the combination of 16 M NaOH solution, the Na_2_SiO_3_/NaOH ratio, and short-term thermal curing, all of which enhanced MK dissolution.

While some MK-based mixtures showed lower oven-dry densities compared to GBFS-based mixtures, this does not contradict their higher compressive strength. Oven-dry density depends not only on pore volume but also on the specific gravity of the precursor and the aggregate content. Because GBFS has a higher specific gravity than MK, GBFS-based mortars might display higher densities even if their matrix contains microcracks or a less continuous gel network. Thus, density alone does not directly reflect mechanical performance in multi-precursor geopolymers [56].

The increased strength of MK-based mixtures, especially under the 16 M–90 °C condition, can be attributed to the high reactivity of metakaolin and the development of a more continuous aluminosilicate gel network. Apparent porosity, measured through water absorption, mainly indicates open and permeable voids but does not fully capture pore-size distribution, closed gel pores, pore connectivity, or gel cohesion. Therefore, compressive strength depends not only on pore quantity but also on the continuity of reaction products, pore connectivity, microcracks, and the bonding capacity of the geopolymer gel [35,48]. In this study, the MK–16M–90 °C mixture showed low apparent porosity and a well-developed aluminosilicate matrix, leading to the highest compressive strength. In contrast, the higher density of GBFS-based mixtures was partly due to GBFS’s higher specific gravity and did not necessarily reflect better matrix quality.

WBP-based mixtures yielded the lowest or near-lowest strength values across all ages. Under 12 M–60 °C conditions, the strength of WBP was 2.1 MPa after 1 day, 3.5 MPa after 7 days, and 4.0 MPa after 28 days. At 12 M–90 °C, the 28-day strength increased to 7.9 MPa, and at 16 M–90 °C, it increased to 9.5 MPa. This increase indicates that high temperature and high alkalinity partially activate the limited reactive phases in WBP. However, it is expected that WBP will produce lower strength than FA, GBFS, and MK because it has a coarser particle size, a more crystalline mineralogical structure, and low-reactive phases such as quartz and mullite. It has been reported that in waste brick dust-based alkali-activated systems, strength largely depends on the fineness of the brick dust, the amount of amorphous phase, the activator concentration, and other co-additives; however, achieving high strength in systems based solely on WBP is limited [57,58]. Therefore, the fact that WBP strength remains low despite improvement at 16 M–90 °C is consistent with the high porosity and water absorption observed in the physical properties.

When assessing age-related strength development, it is evident that early strength is quite prominent in the GBFS and MK–16 M systems. The GBFS–12M–60 °C sample gained 16.2 MPa after one day and reached only 17.6 MPa by 28 days, indicating that most of the reaction occurred within the first 24 h. Similarly, the MK–16M–90 °C sample achieved strengths of 22.5 MPa in 1 day, 23.0 MPa after 7 days, and 25.8 MPa after 28 days. Conversely, the strength increase is more gradual in the FA and WBP samples. For instance, the FA–16M–90 °C sample had a strength of 15.8 MPa after 1 day, 16.9 MPa after 7 days, and 19.9 MPa after 28 days. The WBP–16M–90 °C sample’s strength rose from 5.9 MPa after 1 day to 9.5 MPa in 28 days. This reveals that rapid gel formation predominates in slag and sufficiently activated MK systems, whereas the dissolution-polymerization process in FA and WBP systems proceeds more slowly.

When physical properties and mechanical strength are considered together, a clear cause-and-effect relationship emerges. In the previous section, the MK–16M–90 °C mixture exhibited the lowest apparent porosity (6.1%) and water absorption (5.4%), and here it yielded the highest 28-day compressive strength of 25.8 MPa. Similarly, in the FA–16M–90 °C sample, as porosity and water absorption decreased, strength increased to 19.9 MPa. In contrast, the WBP samples exhibited high porosity and water absorption, resulting in low compressive strength. This relationship demonstrates that strength in geopolymer matrices is determined not only by gel chemistry but also by the gel products’ ability to fill voids, the connectivity of capillary pores, and the homogeneity of the matrix [59,60].

**Figure 6:**
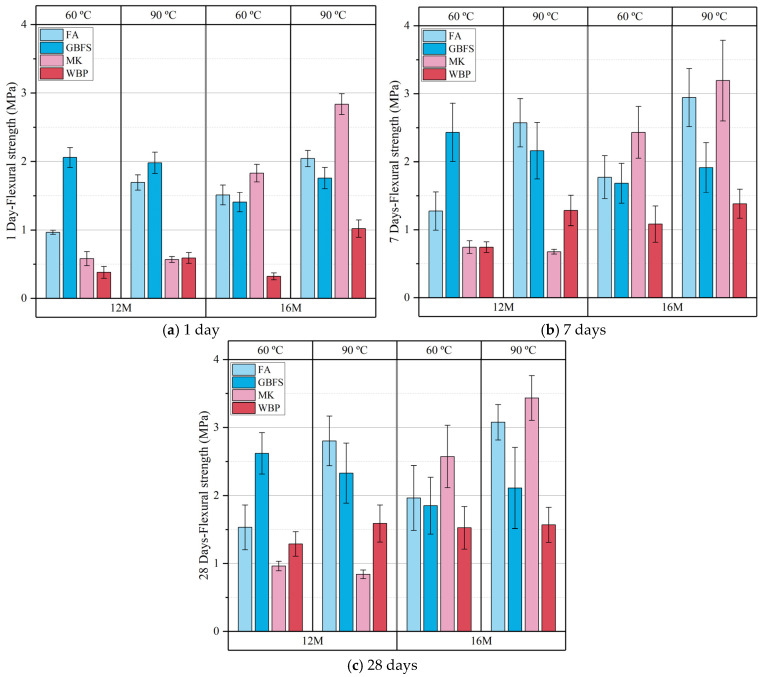
The flexural strength of geopolymer samples.

The flexural strength results shown in Figure 6 are generally consistent with the compressive strength results, although they reveal more sensitive and distinct behavior in certain binder systems. Because flexural strength is more sensitive than compressive strength to defects such as microcracks, interfacial discontinuities, void connectivity, and matrix brittleness, it depends not only on the amount of gel but also on the gel network’s continuity and its resistance to crack propagation.

In FA-based specimens, flexural strength notably increased with higher curing temperatures and NaOH molarity. Under 12 M–60 °C conditions, flexural strengths at 1, 7, and 28 days were 0.97, 1.27, and 1.53 MPa, respectively, whereas at 12 M–90 °C, these values increased to 1.69, 2.57, and 2.81 MPa, respectively. Similarly, at 16 M, the 28-day strength was 1.96 MPa at 60 °C but increased to 3.08 MPa at 90 °C. This trend results from higher alkalinity and temperature, which enhance Si–Al dissolution in low-calcium geopolymers, thereby strengthening the N-A-S-H gel network. Due to FA’s low CaO content, the initial reaction rate is limited; thus, curing at 90 °C produces a notable activation effect, especially in the first 7 days. The literature indicates that curing temperature, time, and alkali concentration significantly influence the mechanical performance of fly ash-based geopolymers, with high-temperature curing accelerating early-age gel formation [25,61]. Therefore, the fact that 16 M–90 °C yields the highest flexural strength aligns with the low porosity and minimal water absorption seen in previous physical tests.

In GBFS-based specimens, the highest flexural strength was observed at 12 M–60 °C, similar to the compressive strength results. The flexural strengths at 1, 7, and 28 days measured 2.06, 2.43, and 2.62 MPa, respectively. Conversely, at 16 M-60 °C, the 28-day strength was 1.85 MPa, and at 16 M–60 °C, it was 2.11 MPa. This indicates that GBFS facilitates rapid formation of C-A-S-H/C-(N)-A-S-H gels, even under milder activation conditions, owing to its high CaO content. However, high molarity combined with high temperature does not always yield better flexural performance in GBFS systems. The likely causes are uneven gel distribution due to overly rapid reactions, microcracks caused by drying, and matrix embrittlement. Previous reports suggest that C-S-H/C-A-S-H-type products support early strength development in alkali-activated slag systems; still, activator dosage and curing conditions critically affect gel microstructure and crack formation [62,63]. Consequently, the 12 M–60 °C condition for GBFS appears to foster a more balanced microstructure, optimizing the response between reaction rate and crack sensitivity.

Specimens based on MK showed the strongest dependence of flexural strength on molarity. At 12 M conditions, MK specimens had relatively low strength, with 28-day measurements of 0.96 MPa at 12 M–60 °C and 0.84 MPa at 12 M–90 °C. Increasing the concentration to 16 M resulted in a sharp increase in flexural strength; at 28 days, it reached 2.57 MPa at 16 M–60 °C and 3.43 MPa at 16 M–90 °C—the highest among all tested series. This suggests that although MK has high Al_2_O_3_ and SiO_2_ content, very fine grains, and an amorphous or dehydroxylated structure, the reaction potential cannot be fully exploited without sufficient alkalinity. Under 16 M–90 °C conditions, MK’s solubility increased, leading to a more continuous and dense aluminosilicate gel network and a matrix better able to resist cracking. Rovnaník showed that curing temperature significantly influences the development of the hardened structure and mechanical properties in metakaolin-based geopolymers [58]. The fact that the 16 M–90 °C mixture of MK has the lowest porosity, and highest flexural strength indicates that matrix density and gel continuity directly control flexural performance in the MK system.

While the flexural strength of WBP-based specimens generally remained low, it showed a more consistent age-dependent trend than the compressive strength. Under 12 M–60 °C conditions, the 1-day flexural strength of WBP was 0.38 MPa, while the 28-day value rose to 1.29 MPa. Under 12 M–90 °C conditions, the 28-day strength was measured at 1.59 MPa, and under 16 M–90 °C conditions, it was 1.57 MPa. These results indicate that curing at 90 °C and high molarity partially activated the limited reactive phases of WBP; however, WBP’s crystalline quartz/mullite phases and coarser grain size prevented the formation of a high-strength gel network. It has been reported that in waste brick dust-based alkali-activated systems, mechanical performance depends largely on the fineness of the brick dust, the amorphous phase ratio, and the activator concentration. In contrast, more crystalline and coarser WBP can limit the degree of reaction [57,64]. Consequently, although the flexural strength of WBP specimens increased up to 28 days, their performance lagged behind that of the optimum mixtures of MK, FA, and GBFS due to high porosity and water absorption.

From an age-dependent development perspective, most of the flexural strength in the GBFS and MK–16 M systems form early. For example, the GBFS–12M–60 °C sample reached 2.06 MPa after 1 day and 2.62 MPa after 28 days. The MK–16M–90 °C sample had strengths of 2.84 MPa after 1 day, 2.84 MPa after 7 days, and 3.43 MPa after 28 days. These findings suggest that strong gel formation occurs early in these systems. Conversely, strength increases more gradually in the FA and WBP samples. Notably, the FA–16M–90 °C sample reached 2.04 MPa at 1 day, 2.94 MPa at 7 days, and 3.08 MPa at 28 days, indicating that the development of N-A-S-H gel over time enhances flexural strength. However, in WBP, 28-day values stayed low due to limited reactivity.

When evaluated alongside the physical properties, the flexural strength results are directly related to the matrix density and pore structure. Among the previous physical properties, the MK–16M–90 °C mixture exhibited the lowest apparent porosity and water absorption; it also yielded the highest 28-day flexural strength, at 3.43 MPa. The FA–16M–90 °C mixture also reached 3.08 MPa, consistent with its low porosity trend. In contrast, the WBP mixtures exhibited low flexural strength due to high porosity and water absorption. This relationship demonstrates that flexural strength is particularly sensitive to open pores, microcracks and weak interface regions. Kan and colleagues reported that curing conditions in alkali-activated fiber composites are decisive for microcrack formation, pore structure and mechanical performance; high-temperature or low-humidity conditions may limit strength by increasing microcrack development in some systems [45]. Therefore, the flexural strength results should be explained not only by the total reaction products but also by the extent to which these reaction products can limit crack propagation.

### 3.3. Transport Properties

**Figure 7:**
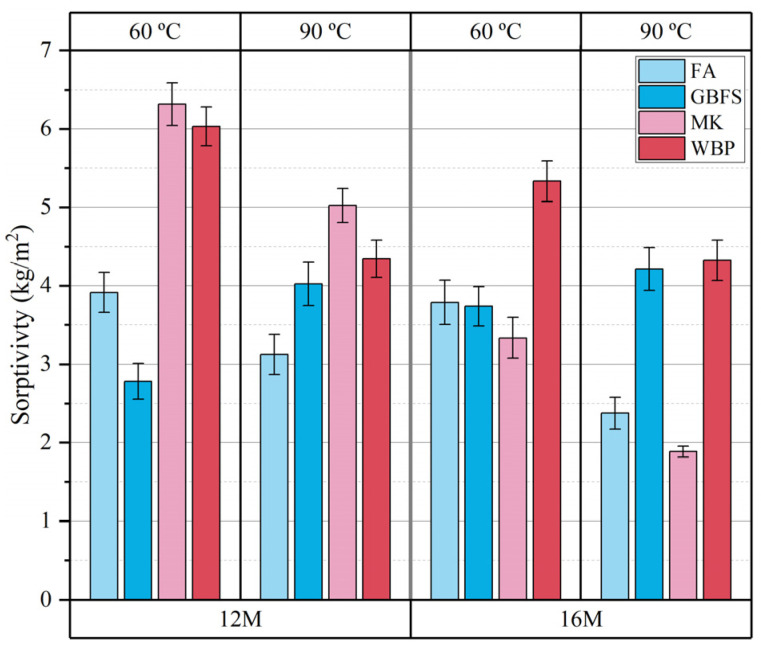
Capillary water absorption of geopolymer samples.

The capillary water absorption results shown in Figure 7 are highly consistent with previous data on apparent porosity, water absorption, and compressive and flexural strength. Capillary water absorption is one of the most critical indicators of the geopolymer matrix’s durability, as it reflects not only the total void content but, in particular, the open, interconnected, and continuous capillary pore network. The literature also indicates that large capillary voids govern sorptivity/permeable water absorption during initial water transport, whereas smaller gel pores govern the absorption rate over time [28,44]. Therefore, the results in the figure should be evaluated not merely in terms of ‘absorbing more or less water’, but through the lens of gel formation, pore refinement, degree of reaction, microcrack formation, and binder reactivity.

In FA-based samples, capillary water absorption generally decreased with the combination of 90 °C curing and 16 M NaOH. While the FA sample had a capillary water absorption of 3.91 kg/m^2^ at 12 M–60 °C, it fell to 3.12 kg/m^2^ at 12 M–90 °C. Similarly, while the value was 3.79 kg/m^2^ at 16 M–60 °C, it reached 2.38 kg/m^2^ at 16 M–90 °C, the lowest value in the FA series. This trend can be explained by the more effective dissolution of low-calcium FA at higher temperatures and sufficient alkalinity. In FA-based systems, N-A-S-H gel formation is accelerated, particularly by thermal curing; the reaction products fill interconnected capillary voids, making the water-transport pathways more tortuous and discontinuous [25,44]. In a study by Noushini and Castel on low-calcium fly ash-based geopolymers, the sorptivity coefficient was also found to decrease under appropriate thermal curing conditions, which is associated with a denser geopolymer network structure and a more refined pore system [44]. In this context, the low capillary absorption exhibited by the FA–16M–90 °C mixture is consistent with the results from the previous section on low porosity, low water absorption, and high compressive/flexural strength.

In contrast to FA, higher molarity and temperature did not reduce capillary water absorption in GBFS-based specimens; in fact, they increased it. The lowest GBFS capillary water absorption, 2.78 kg/m^2^, occurred under 12 M–60 °C conditions. In contrast, the value rose to 4.02 kg/m^2^ at 12 M–90 °C, to 3.73 kg/m^2^ at 16 M–60 °C, and to 4.21 kg/m^2^ at 16 M–90 °C. This result suggests that, due to its high CaO content, GBFS can facilitate sufficient C-A-S-H/C-(N)-A-S-H gel formation even under moderate activation conditions; however, the combination of high temperature and high alkalinity may lead to rapid reactions, water loss, possible moisture-loss-related microcracking, and associated pore development. In alkali-activated slag systems, whilst the Ca content can increase the pore-filling capacity of the gel products, high temperature can induce microcracks and open-pore connectivity within the matrix if the curing regime is not appropriately selected [65]. Therefore, the 12 M–60 °C condition for GBFS appears to yield the most balanced microstructure for capillary water transport.

The capillary water absorption behavior of MK-based samples closely aligns with earlier physical and mechanical results. At 12 M–60 °C, the MK sample exhibited one of the highest absorption rates among all series, reaching 6.32 kg/m^2^. Although this decreases to 5.02 kg/m^2^ at 12 M–90 °C, it remains relatively high. A notable improvement occurs when the NaOH molarity increases to 16 M; at 16 M–60 °C, absorption drops to 3.34 kg/m^2^, and further to 1.88 kg/m^2^ at 16 M–90 °C. This is the lowest absorption among all mixes. Despite MK’s high Al_2_O_3_ and SiO_2_ content, very fine particles, and amorphous/dehydroxylated structure, it could not form a dense gel network at 12 M activation. However, under 16 M–90 °C conditions, increased dissolution and polycondensation occurred. Research shows that in metakaolin-based geopolymers, alkali concentration and curing regime influence the dissolution-polymerization balance, leading to a denser, less permeable matrix [66,67]. The lowest porosity, highest mechanical strengths, and minimal capillary water absorption in the MK–16M–90 °C sample indicate significant development of gel continuity and pore refinement.

Capillary water absorption in WBP-based samples remained generally high. Under 12 M–60 °C conditions, WBP showed a capillary water absorption of 6.05 kg/m^2^. This value decreased to 4.34 kg/m^2^ at 12 M–90 °C, was 5.33 kg/m^2^ at 16 M–60 °C, and reached 4.33 kg/m^2^ at 16 M–90 °C. Although the 90 °C curing treatment significantly improved the WBP samples, the values remain higher than those under the optimum conditions for FA and MK. This behavior can be explained by WBP’s coarser particle size, more crystalline structure, and the presence of low-reactive phases such as quartz and mullite. It has been reported that in waste clay brick dust-based alkali-activated systems, reactivity depends largely on fineness, the amorphous phase ratio, and activator conditions; WBP that is not sufficiently fine and reactive may exhibit a partially inert/filler character within the matrix, potentially failing to seal the interconnected pore system [18,32] fully. Hwang and colleagues demonstrated that the performance of high-volume WBP can be improved when blended with slag, but that the degree of reaction may remain limited when WBP is used alone or at high crystallinity [18] Therefore, in the present study, the partial improvement in WBP at 90 °C can be explained by increased activation of limited reactive phases; however, the persistence of high capillary absorption suggests that the crystalline/coarse structure cannot completely sever pore connectivity.

The results regarding NaOH molarity are inconsistent. While 16 M NaOH reduced capillary water absorption in the MK and FA systems, it did not have the same positive effect in the GBFS and WBP systems. This is significant because the generalization that ‘durability always improves as molarity increases’ does not hold for this dataset. Ilayarsi and colleagues reported that NaOH molarity can accelerate geopolymerization to an optimal level; however, excessive molarity may reduce workability and increase the risk of rapid reaction and microcracking [23]. Pratap and colleagues also noted that increasing NaOH molarity reduces porosity, water absorption, and sorptivity to an optimal level; however, if this level is exceeded, stability indicators may deteriorate again due to excessive alkalinity and unstable gel formation [68]. In this study, while 16 M appears necessary and favorable for MK, the 12 M–60 °C condition is more successful for GBFS, clearly demonstrating that the optimum molarity depends on the binder chemistry.

When evaluated alongside the physical and mechanical properties, capillary water absorption values generally show a positive correlation with porosity and water absorption and an inverse correlation with compressive and flexural strength. For example, the MK–16M–90 °C sample exhibited the lowest apparent porosity and water absorption values, and the highest compressive and flexural strengths in previous sections; it also has the lowest capillary water absorption value here, at 1.88 kg/m^2^. In contrast, high-porosity mixtures such as WBP–12M–60 °C and MK–12M–60 °C exhibited high capillary water absorption. Zhu et al. reported strong correlations among compressive strength, sorptivity, and pore structure in geopolymer systems, and that an increase in strength generally occurs alongside a decrease in open-pore volume and water transport [60]. However, capillary water absorption should not be used as the sole indicator of ultimate durability. Noushini and Castel emphasized that sorptivity, permeable void volume, and compressive strength in geopolymer concretes do not, on their own, reliably reflect chloride diffusion performance; therefore, capillary water absorption results should be evaluated alongside transport tests such as electrical resistivity and chloride permeability [28].

**Figure 8:**
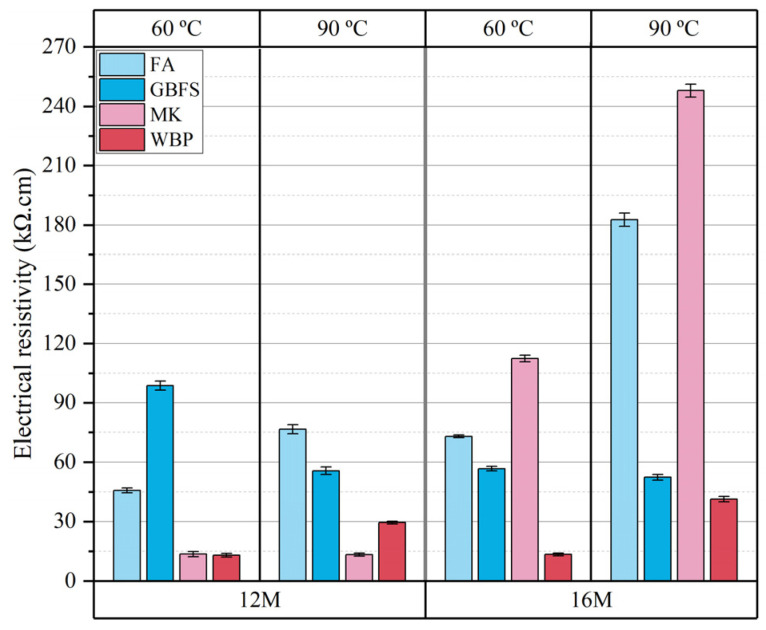
Electrical resistivity properties of geopolymer samples.

Evaluating the electrical resistivity results in Figure 8 alongside earlier data on physical, mechanical, and capillary water absorption reveals that ionic transport resistance in geopolymer matrices depends on factors beyond total porosity. It also involves the interconnected pore structure, the free-ion concentration in the pore solution, gel continuity, and the binder type. Measurements were conducted on 40 × 40 × 160 mm prisms under SSD conditions per ASTM C1876 [43], so the values reflect ionic conductance in a saturated matrix rather than in dry conditions. The literature indicates that the electrical resistivity of geopolymers should be interpreted differently from that of Portland cement systems, because mobile ions such as Na^+^, K^+^, and OH^−^ in the pore solution can enhance conductivity even in dense microstructures [28,44]. Noushini and Castel found that low-calcium FA-based geopolymers cured thermally exhibit reduced permeability, voids and capillary continuity, thereby increasing both electrical resistivity and compressive strength [44]. They highlighted that low electrical resistivity may be related not only to porosity but also to free Na^+^ ions in the pore solution.

In FA-based samples, electrical resistivity increased significantly with higher molarity and curing temperature. Under 12 M–60 °C conditions, the resistivity of the FA sample was 45.67 kΩ·cm, whereas at 12 M–90 °C it increased to 76.54 kΩ·cm. Similarly, a value of 72.93 kΩ·cm was obtained at 16 M–60 °C, whilst at 16 M–90 °C it reached 182.67 kΩ·cm. This increase is consistent with previous physical results showing decreased porosity and water absorption in FA mixtures, as well as mechanical results showing increased strength. In low-calcium FA-based geopolymers, higher temperatures and alkalinity accelerate the formation of N-A-S-H gels by increasing the solubility of Si and Al species; these gel products fill interconnected capillary voids, making the ion-transport pathways less continuous and more tortuous [44,69]. Consequently, the high electrical resistivity in the FA–16M–90 °C sample indicates the formation of a denser and more continuous aluminosilicate network structure in this mixture. However, in FA systems, electrical resistivity depends not only on the pore structure but also on the binding and migration of free alkali ions from the activator over time [28].

The trend in GBFS-based samples is markedly different from that of FA. The highest GBFS resistivity, 98.67 kΩ·cm, was observed at 12 M and 60 °C. However, at 12 M–90 °C, the value dropped to 55.65 kΩ·cm; under 16 M–60 °C and 16 M–90 °C conditions, it remained at 56.75 and 52.35 kΩ·cm, respectively. This suggests that, due to its high CaO content, GBFS can form rapid, dense C-A-S-H/C-(N)-A-S-H-type gel products under moderate activation conditions; however, the combination of high temperature and high alkalinity may adversely affect pore solution chemistry and microcrack development. In previous capillary water absorption results, the lowest GBFS value was obtained at 12 M–60 °C, whilst capillary absorption increased at higher temperatures and molarities. Therefore, the high resistivity in the GBFS–12M–60 °C mixture can be attributed to a less interconnected capillary pore system. Conversely, the decrease in resistivity under 16 M conditions can be explained by a more concentrated alkaline environment, which increases the number of free ions and/or microcracks formed by rapid reactions, thereby facilitating ionic conduction pathways. Xi and colleagues noted that the precursor type, alkali concentration, curing temperature, and slag content significantly influence the electrical behavior in geopolymers; whilst slag-rich systems can form a denser matrix due to C-(A)-S-H-type products, the presence of ions and the conductivity of the pore solution are important factors affecting electrical resistivity [70].

MK-based samples exhibited the most pronounced electrical resistivity behavior. Under 12 M conditions, the resistivity of MK samples was quite low, measured at 13.58 kΩ·cm at 12 M–60 °C and 13.53 kΩ·cm at 12 M–90 °C. In contrast, a sudden and very strong increase in resistivity occurred upon switching to 16 M; 112.46 kΩ·cm was obtained at 16 M–60 °C, and 248.00 kΩ·cm at 16 M–90 °C, the highest value in the entire series. This result is highly consistent with all previous data. The MK–16M–90 °C mixture previously had the lowest apparent porosity, water absorption and capillary water absorption and the highest compressive/flexural strength. Therefore, in this mixture, not only is the total void volume reduced, but the interconnected capillary pores are largely disrupted. The very fine-grained structure and high Al_2_O_3_–SiO_2_ content of MK allowed it to form a dense aluminosilicate gel network when sufficient alkalinity was provided. In the study by Cai et al. on electrical resistivity in FA- and MK-based geopolymers, it was also reported that the precursor type, alkali concentration, and curing conditions are decisive factors [30]. In addition, the high degree of reaction in metakaolin-based systems can increase gel continuity and limit ionic conduction pathways; this explains the very high resistivity of the MK–16M–90 °C sample in the present study.

Electrical resistivity in WBP-based samples generally remained low. The resistivity of WBP was 13.07 kΩ·cm at 12 M–60 °C and increased to 29.46 kΩ·cm at 12 M–90 °C. At 16 M–60 °C, the value remained at 13.44 kΩ·cm, and at 16 M–90 °C, it reached 41.52 kΩ·cm. These results show that curing at 90 °C significantly improves the WBP system, but increasing molarity alone is insufficient. Due to its coarser grain size, crystalline quartz/mullite phases, and limited amorphous content, the degree of geopolymerization in WBP remains lower than in FA and MK. Consequently, complete closure of open or connected pore structures in the WBP matrix is difficult, and ionic conduction pathways persist. The higher resistivity at 16 M–90 °C suggests that high temperature partially activates the limited reactive phases, thereby increasing gel formation; however, the values remain considerably lower than under the optimum conditions for MK and FA, indicating that the crystalline/coarse character of WBP limits its transport resistance. Various studies have reported that the performance of WBP-based alkali-activated systems is sensitive to fineness, the amount of amorphous phase, and activator conditions; high crystallinity and low solubility can negatively affect the pore structure [71,72].

A key scientific insight from these results is that the mechanisms controlling electrical resistivity vary with binder type. In FA and MK systems, the 16 M–90 °C condition notably raised resistivity, which correlates with low capillary absorption, low porosity, and high strength. Conversely, the optimal condition for GBFS was 12 M–60 °C. This indicates that more aggressive curing or activation conditions in high-Ca slag systems do not necessarily enhance transport resistance. In WBP, although high temperatures are advantageous, resistivity remains limited due to crystalline structure and coarse grains. Consequently, electrical resistivity cannot be universally explained by simple rules such as “resistivity increases with molarity” or “transport resistance improves with temperature.” Instead, a comprehensive evaluation of each binder’s reactivity, gel chemistry, pore solution, and pore connectivity is essential for accurate interpretation.

There is generally an inverse relationship between electrical resistivity and capillary water absorption. The MK–16M–90 °C sample exhibited the lowest capillary water absorption, 1.88 kg/m^2^, and the highest resistivity, 248.00 kΩ·cm. The FA–16M–90 °C sample also showed low capillary absorption and high resistivity. In contrast, the WBP–12M–60 °C and MK–12 M conditions produced low resistivity and high capillary water absorption, or a tendency toward high porosity. This relationship indicates that resistivity is sensitive to the capillary pore system with which it is associated. However, in geopolymer systems, resistivity alone should not be used to assess permeability, as free Na^+^/OH^−^ ions in the pore solution can reduce resistivity. Górski et al. demonstrated that the electrical response in geopolymers is related to ionic conduction and capacitive behavior, and that mobile ions in pores play an important role in electrical measurements [73]. Similarly, pore structure and pore solution chemistry should be considered together in RCPT and resistivity interpretations [74,75].

It is important to note that ASTM C1202 [27] does not directly assess the chloride diffusion coefficient or true chloride permeability, especially in alkali-activated and geopolymer systems. The total charge passed can be affected not only by pore structure but also by the ionic conductivity of the pore solution, which includes mobile Na^+^, K^+^, and OH^−^ ions from the alkaline activator. As a result, this study uses ASTM C1202 [27] results as a comparative indicator of chloride-ion penetrability and electrical transport, rather than as an exact measure of permeability. The transport performance of the mixtures was evaluated alongside capillary water absorption, electrical resistivity, apparent porosity, water absorption, and SEM/EDS observations to avoid relying solely on ASTM C1202 [27] classification.

**Figure 9:**
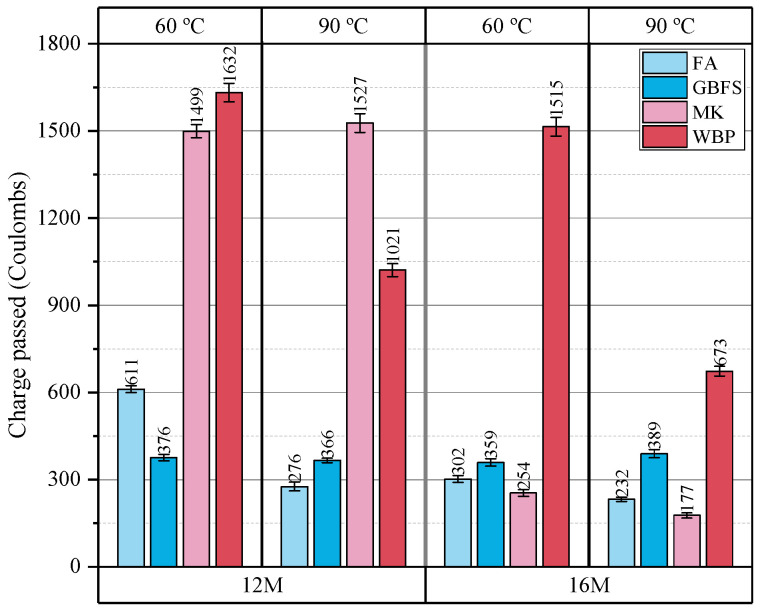
Chloride-ion penetrability properties of geopolymer samples.

The total charge-passage results for ASTM C1202 [27], shown in Figure 9, indicate that the samples’ resistance to chloride-ion passage varies significantly with binder type, NaOH molarity, and curing temperature. The key point is that the value measured in the ASTM C1202 [27] test is not “chloride-ion penetrability” alone, but rather the total electrical charge passing through the specimen. Therefore, in geopolymers, these results must be evaluated in conjunction with the conductivity of mobile ions such as Na^+^ and OH^−^ in the pore solution, the interconnected pore structure, gel continuity, and matrix density.

Chloride-ion penetrability in FA-based samples decreased significantly with increasing molarity and curing temperature. The FA sample had a permeability of 611 C under 12 M–60 °C conditions and dropped to 276 C at 12 M–90 °C. Similarly, values of 302 C were obtained at 16 M–60 °C and 232 C at 16M–90 °C. According to the ASTM C1202 [27] classification, all these values fall into the “very low chloride ion penetrability” category; however, the 16 M–90 °C condition provided the lowest chloride ion flux. This result aligns with the previous physical and transport properties: the FA–16M–90 °C sample exhibited lower porosity, lower capillary water absorption, and higher electrical resistivity. In low-calcium FA systems, high temperature and sufficient alkalinity enhance the dissolution of Si and Al, thereby promoting N-A-S-H gel formation; this gel structure limits ion transport by reducing interconnected capillary pathways [29,76]. Chindaprasirt and Chalee also reported that the NaOH concentration in FA-based geopolymer concrete is a determining factor in chloride penetration and reinforcement corrosion. That chloride migration can be limited under appropriate activation conditions [29].

In GBFS-based samples, the total permeate load values are similar and low across all conditions. The values obtained were 376 C at 12 M–60 °C, 366 C at 12 M–90 °C, 359 C at 16 M–60 °C, and 389 C at 16 M–90 °C. All of these values fall into the “very low chloride-ion penetrability” class according to ASTM C1202 [27]. Although previous electrical resistivity results for GBFS showed higher resistivity under 12 M–60 °C conditions, all GBFS mixtures must remain similar in the RCPT results. This indicates that GBFS generally forms a dense, low-permeability matrix due to C-A-S-H/C-(N)-A-S-H-type gel formation resulting from its high CaO content; however, total ion transport is controlled not only by the pore structure but also by the ionic conductivity of the pore solution. Ismail and colleagues reported that FA content significantly affects the penetrability of water and chloride ions in alkali-activated slag mortars and concretes; they noted that chloride transport in slag-based systems must be evaluated in conjunction with gel structure, pore continuity, and binder chemistry [77]. Yang and colleagues also demonstrated that chloride diffusion in FA–slag-based geopolymers is influenced not only by the number of voids but also by reaction products and microstructural continuity [78].

The most dramatic change in MK-based samples occurred with NaOH molarity. Under 12 M–60 °C and 12 M–90 °C conditions, the charge passing through the MK samples was 1499 C and 1527 C, respectively. Although these values fall into the “low chlorine permeability” class per ASTM C1202 [27], they are still quite high compared to other binders. In contrast, when the concentration was increased to 16 M, the chlorine permeability of MK decreased sharply; values of 254 C at 16 M–60 °C and 177 C at 16 M–90 °C were obtained. This result strongly aligns with all previous data. The MK–16M–90 °C mixture exhibited the lowest porosity, water absorption, and capillary water absorption, and the highest compressive/flexural strength and electrical resistivity. Consequently, under the 16 M–90 °C conditions, MK’s high Al_2_O_3_ and SiO_2_ content dissolved effectively with sufficient alkalinity and thermal energy, forming a dense and continuous aluminosilicate gel network, and the interconnected pore system through which chloride ions could migrate was largely disrupted. In contrast, under 12 M conditions, MK’s high reactivity potential was not fully utilized; due to a more open pore structure and lower resistivity, high ion transport occurred. This demonstrates that selecting the activator concentration at an optimal level in geopolymers is critical not only for strength but also for transport properties [30,79].

Samples based on WBP typically showed the highest chlorine permeability values. At 12 M–60 °C, the flux through the WBP sample reached 1632 C, the highest among all series. At 12 M–90 °C, this decreased to 1021 C, yet it still fell within the “low chlorine permeability” category. Under 16 M–60 °C conditions, WBP again exhibited a high value of 1515 C. Conversely, at 16 M–90 °C, the total charge decreased to 673 C, entering the “very low chlorine permeability” range. This suggests that WBP has difficulty forming a dense gel network due to its coarser particles, its more crystalline mineral structure, and the presence of low-reactive phases such as quartz and mullite. However, the 90 °C curing temperature partially activated WBP’s limited reactive phases, reducing chlorine permeability. Despite this, WBP’s higher permeability compared to optimal FA and MK conditions aligns with previous findings of high porosity, capillary absorption, and low electrical resistivity. Rakhimova and Rakhimov demonstrated that performance in alkali-activated systems with red clay brick waste is largely influenced by slag co-use, reaction degree, and microstructural densification [72]. Therefore, it is expected that WBP will exhibit less chlorine resistance when used as a standalone binder.

When combined with the electrical resistivity data, these findings demonstrate a clear inverse relationship. The MK–16M–90 °C sample, which had the highest electrical resistivity at 248 kΩ·cm, showed a charge transfer of only 177 C. Similarly, the FA–16M–90 °C sample displayed high resistivity and low charge transfer. Conversely, WBP–12M–60 °C and WBP–16M–60 °C samples had low resistivity but high Coulomb values. This suggests that the pore structure’s interconnectedness and ion transport pathways are crucial for chloride migration. However, the fact that the resistivity and RCPT results for the GBFS samples do not always follow the same order indicates that pore-solution chemistry significantly influences total charge transfer. Ravikumar and Neithalath noted that chloride transport driven by electrical means in alkali-activated blast furnace slag concretes depends on both microstructure and ionic conductivity [80]. Consequently, especially in geopolymers, ASTM C1202 [27] results should not be viewed in isolation. Instead, they should be considered alongside porosity, capillary absorption, electrical resistivity, and, if available, actual chloride diffusion profiles [80,81].

Table 5 presents an evaluation of the samples’ performance based on the classification of chlorine permeability and electrical resistivity as defined by ASTM C1202 [27] and Spragg et al. [82]. According to the classification table, all geopolymer samples fell into the “low” or “very low” categories for chloride ion penetrability, and no mixture exhibited “medium” or “high” permeability. For electrical resistivity, no sample posed a “high” or “very high” corrosion risk. All FA-based mixtures, with resistivity values ranging from 45.67 to 182.67 kΩ·cm and charge transfer rates ranging from 232 to 611 C, fell into the “negligible corrosion risk” and “very low chloride-ion penetrability” categories, respectively. GBFS-based mixtures also fell into the “negligible corrosion risk” and “very low chloride-ion penetrability” classes under all conditions; this was attributed to GBFS forming dense C-A-S-H/C-(N)-A-S-H gel products due to its high Ca content. In MK-based mixtures, 12 M activation was insufficient, and the samples remained in the “low corrosion risk/low chloride-ion penetrability” class; however, the 16 M–90 °C condition provided the best performance among all series, with a resistivity of 248.00 kΩ·cm and a chloride-ion penetrability of 177 C. In WBP-based samples, the 12 M and 60 °C conditions were relatively weak; however, under the 16 M–90 °C condition, the sample advanced to the “very low chloride-ion penetrability” and “negligible corrosion risk” classes. These results indicate that the dense gel network formed under high reactivity and sufficient activation limits interconnected capillary pores, thereby reducing both electrical ion transport and chloride ion migration.

**Figure 10:**
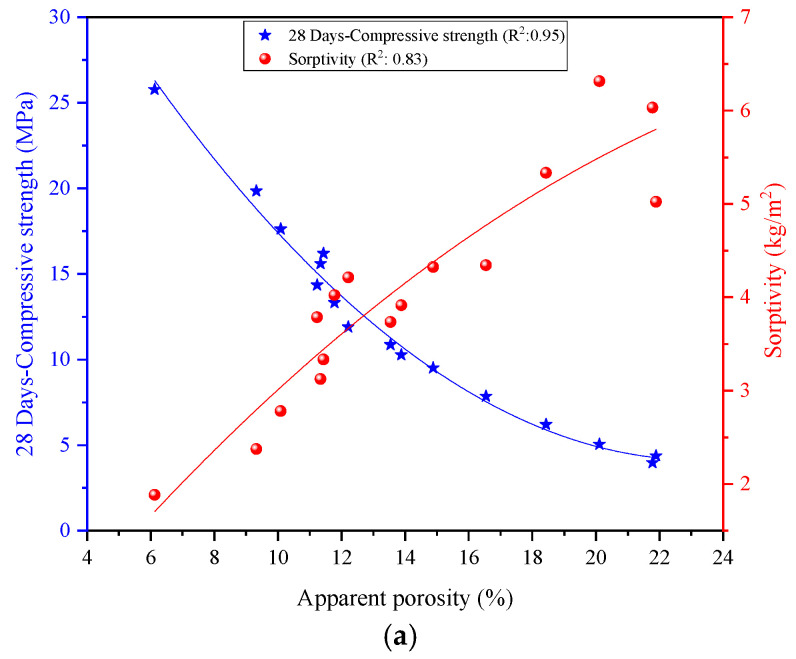

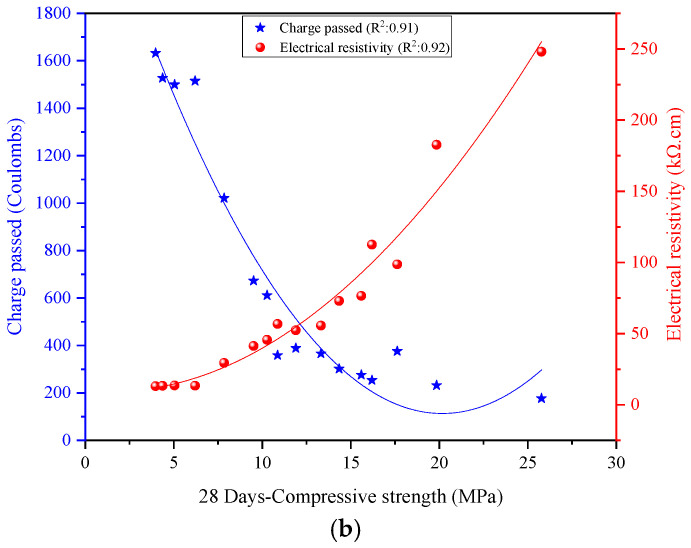
Regression-based correlations between pore-structure-related properties and performance indicators. (**a**) Relationship of apparent porosity with 28-day compressive strength and sorptivity; (**b**) Relationship between 28-day compressive strength, chloride-ion charge passed, and electrical resistivity.

Figure 10 illustrates strong correlations among the physical, mechanical, and transport properties of geopolymer samples. A very strong inverse correlation exists between apparent porosity and 28-day compressive strength (R^2^ = 0.95), indicating porosity’s crucial role in mechanical performance. Higher porosity reduces the load-bearing solid phase, promotes microcracking, and weakens the material [68]. Conversely, a positive correlation (R^2^ = 0.83) was found between apparent porosity and capillary water absorption, showing that water uptake depends not only on the total void volume but also on void connectivity and pore structure [49]. There is also a strong inverse relationship (R^2^ = 0.91) between compressive strength and ASTM C1202 [27] total passing load, as chloride ion transfer is impeded in higher-strength samples due to a denser gel network and less interconnected porosity. Nevertheless, since mobile alkali ions can also influence chloride-ion penetrability in the pore solution, this correlation should not be seen as a direct measure of chloride diffusion [44]. Lastly, a strong positive correlation (R^2^ = 0.92) was observed between 28-day compressive strength and electrical resistivity, suggesting that denser, more continuous geopolymer matrices restrict ion pathways and lower electrical conductivity [73]. Overall, these correlations demonstrate that low porosity and capillary absorption are linked to superior mechanical strength, higher electrical resistivity, and lower chloride-ion penetrability.

### 3.4. Cost Analysis

A cost analysis was conducted based on the quantities of materials used to prepare geopolymer mixtures. The unit prices of the materials are listed in Table 6. Since NaOH solutions of two different molarities (12 M and 16 M) were used in preparing the mixtures, two different cost parameters were applied. This is because NaOH pellets were used in different proportions in these solutions, and their solid content varies. Geopolymer samples were subjected to thermal curing at 60 and 90 °C. The curing time was the same for both curing conditions (24 h). Because the oven’s electricity consumption was not monitored separately during curing, the cost analysis was based on a simplified sensitivity assumption. The 90 °C curing regime was assigned a 10% higher cost than the 60 °C regime to reflect the additional energy required to reach and maintain the higher temperature over the same 24 h curing period. The cost index was calculated by dividing the production cost per cubic meter by the 28-day compressive strength.

**Figure 11:**
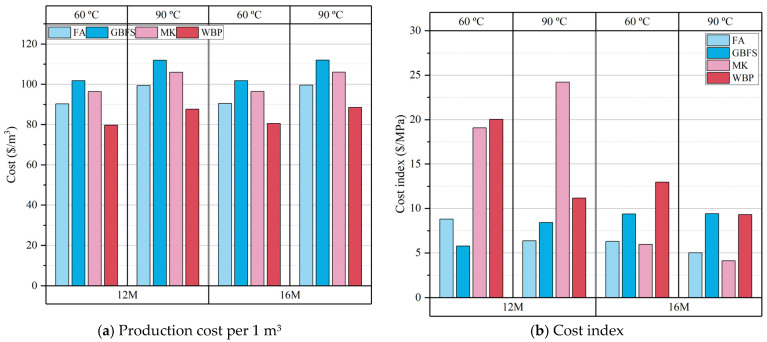
Cost analysis of geopolymer samples.

As shown in Figure 11a, the lowest production cost was observed in WBP-based mixtures across all conditions. In WBP samples, the cost was calculated as 79.7 $/m^3^ at 12 M–60 °C, 87.7 $/m^3^ at 12 M–90 °C, 80.5 $/m^3^ at 16 M–60 °C, and 88.6 $/m^3^ at 16 M–90 °C. This result is expected, as the unit price table shows WBP as the lowest-cost binder at $ 11/ton. In contrast, GBFS-based mixtures had the highest production cost: approximately 101.8 $/m^3^ at 60 °C and 112.0 $/m^3^ at 90 °C. This outcome reflects both the highest unit price among the binders and the large amount of quartz sand required to complete the mixture volume. FA and MK mixtures fell into the intermediate cost group; FA was approximately $90–100/m^3^, while MK was approximately $96–106/m^3^.

Increasing the NaOH molarity from 12 M to 16 M had a limited effect on production costs. For instance, the FA cost was 90.3 $/m^3^ at 12 M–60 °C, and 90.5 $/m^3^ at 16 M–60 °C. Similarly, MK values were nearly identical, at 96.3 $/m^3^ for 12 M–60 °C and 96.4 $/m^3^ for 16 M–60 °C. This is because, although the unit cost of 16 M NaOH was higher, the amounts of the main activator and binder remained constant, and the volumetric adjustment of quartz sand partially offset this difference. Conversely, increasing the curing temperature from 60 °C to 90 °C resulted in a direct cost increase across all mixtures, due to a 10% increase in thermal curing costs. This reflects the higher energy requirement at 90 °C over 24 h. Since activator production and thermal curing are key factors limiting economic feasibility in geopolymers, this cost-sensitive approach is reasonable and justifiable [83].

However, the cost index results in Figure 11b show that focusing solely on the production cost per 1 m^3^ can be misleading. Although WBP has the lowest production cost, it becomes expensive per unit strength in many conditions because of its low compressive strength. For example, although the production cost of the WBP–12M–60 °C mixture is only 79.7 $/m^3^, its cost index is 20.0 $/MPa, one of the least favorable values among all series. Similarly, the cost index of the WBP–16M–60 °C mixture is 13.0 $/MPa. This shows that the low material cost of WBP does not directly translate into structural efficiency because of its limited mechanical performance. The more crystalline structure, coarse-grained distribution, and low reactive-phase content of WBP have previously been considered limiting factors for its mechanical and transport properties. Nevertheless, WBP remains a significant advantage because it is a low-cost, abundant waste source, especially for applications requiring low-to-medium strength or where waste utilization is a priority [49].

The most notable results were observed with MK-based mixtures. Although MK has a higher production cost than WBP, it achieved the lowest cost index of 4.1 $/MPa due to its exceptional compressive strength at 16 M–90 °C. These findings align with previous research, as the MK–16M–90 °C mixture also exhibited the lowest porosity, capillary water absorption, and chloride-ion penetrability, along with the highest compressive and flexural strength and electrical resistivity. Consequently, this mixture is the most efficient system, both technically and cost-effectively. Conversely, the cost index for MK in 12 M conditions is less favorable: 19.1 $/MPa for MK–12M–60 °C and 24.2 $/MPa for MK–12M–90 °C. This indicates that MK’s high reactivity yields an economic benefit only when there is sufficient alkalinity and the temperature is appropriate.

FA-based mixtures perform well in terms of cost-performance balance. The cost index is 8.8 $/MPa for FA–12M–60 °C, decreasing to 6.4 $/MPa for 12 M–90 °C, 6.3 $/MPa for 16 M–60 °C, and 5.0 $/MPa for 16 M–90 °C. This trend indicates that higher alkalinity and 90 °C curing in FA systems improve mechanical performance and reduce the unit strength cost. In particular, the FA–16M–90 °C mixture, although not significantly cheaper than MK–16M–90 °C in production cost, is quite competitive in terms of performance index. Furthermore, previous durability results showed that the FA–16M–90 °C mixture exhibited very low chloride-ion penetrability and high electrical resistivity. Therefore, FA–16M–90 °C can be considered a strong alternative, offering a balance of high technical performance, reasonable cost, and good durability.

The cost behavior of GBFS-based mixtures differs from that of others. Although producing these mixtures is the most expensive among all binders, the GBFS–12M–60 °C mix achieved a highly cost-effective result with a cost index of $5.8/MPa. This is because GBFS can develop high early and final compressive strength under moderate activation conditions. However, under 16 M conditions, GBFS strength decreased while production costs remained similar, resulting in a higher cost index of $9.4/MPa. This indicates that high molarity and high temperature are not economically practical for GBFS. In other words, the 12 M–60 °C condition is more suitable for cost-efficient GBFS use because it avoids additional thermal costs while still delivering adequate mechanical and durability performance. The literature reports that slag and high-calcium precursors can form dense C-A-S-H and C-(N)-A-S-H gels upon proper activation, which are beneficial for early strength development [84].

In a general engineering ranking, WBP is the most cost-effective option based on unit volume, while MK–16M–90 °C offers the best value for unit strength cost. If the goal is solely to lower material costs, WBP mixtures seem advantageous. However, for optimizing overall structural performance—considering strength, low permeability, and cost—the advantage of WBP diminishes. In such cases, MK–16M–90 °C, FA–16M–90 °C, and GBFS–12M–60 °C emerge as the top options, respectively. GBFS–12M–60 °C, notably, may be more practical from an energy perspective since it does not require 90 °C curing. Conversely, MK–16M–90 °C is a more rational choice for applications demanding high strength and durability, as it delivers the highest technical performance. FA–16M–90 °C provides a balanced approach between these priorities.

### 3.5. Microstructure Analysis

In SEM images, phase identification was not solely based on morphology. Instead, it involved integrating SEM observations with local EDS compositions, precursor characteristics, and previously established gel chemistry criteria for alkali-activated materials. In low-calcium systems, such as FA- and MK-based mixtures, gel-like regions rich in Si, Al, Na, and O were identified as N-A-S-H-type aluminosilicate reaction products. In calcium-rich systems like GBFS-based mixtures, areas containing Ca along with Si, Al, and O were classified as C-A-S-H/C-(N)-A-S-H-type or calcium-containing aluminosilicate reaction products. Since SEM/EDS cannot definitively identify crystallographic phases, these labels are considered as ‘gel-like regions’ or ‘N-A-S-H-/C-A-S-H-type reaction products,’ rather than precise phase designations. This terminology more accurately reflects that the interpretation of gels relies on a combination of morphological and microchemical evidence.

#### 3.5.1. SEM Examinations

In this study, “gel formation” and “pore refinement” refer to microstructural features inferred from physical, mechanical, transport, and SEM/EDS analyses. It is important to note that pore size distribution was not directly measured with methods like MIP, BET, or micro-CT. Instead, the enhancement in pore structure was deduced from indicators such as reduced apparent porosity, decreased water absorption and capillary water uptake, increased electrical resistance, decreased charge transfer, and the presence of denser gel-like reaction products observed in SEM images.

**Figure 12:**
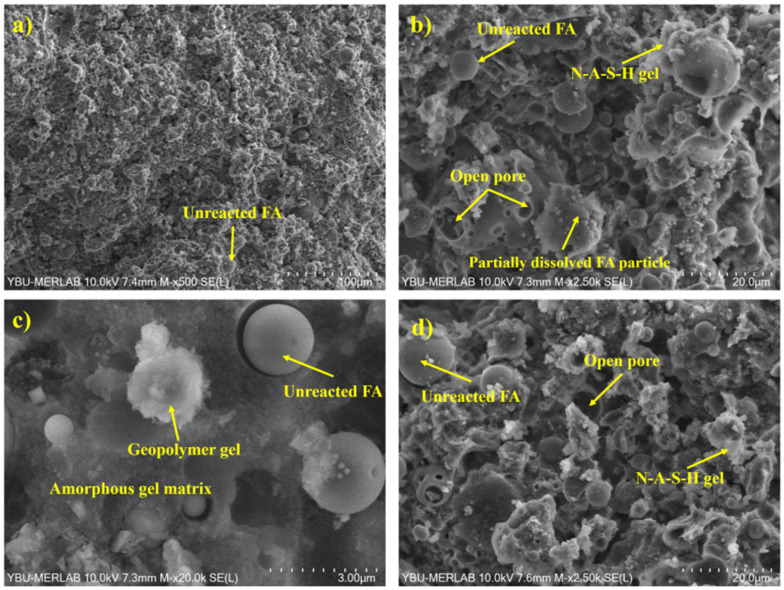
SEM images of the FA-based geopolymer mortar activated with 12 M NaOH and cured at 60 °C. (**a**) General matrix morphology. (**b**) Partially dissolved FA particles and open pores. (**c**) Amorphous geopolymer gel matrix. (**d**) Heterogeneous matrix with N-A-S-H-type gel and open pores.

Figure 12 presents SEM micrographs of the FA-based geopolymer mixture activated with 12 M NaOH and cured at 60 °C. The microstructure shows a heterogeneous and partially reacted matrix with many spherical fly ash particles, open pores, and regions of discontinuous gel. The spherical particles visible especially in Figure 12b–d are typical of fly ash and suggest that a significant portion of the precursor remained unreacted or only partially dissolved under the given activation and curing conditions. Similar findings have been reported for fly ash-based geopolymers, where the presence of discrete unreacted FA particles was linked to incomplete aluminosilicate dissolution and limited geopolymerization [85]. This characteristic aligns with the moderate mechanical and transport properties observed in the FA–12M–60 °C mixture in this study.

The partially dissolved FA particles, surrounded by reaction products, indicate that geopolymerization primarily occurred via the surface dissolution of fly ash spheres, followed by the formation of an aluminosilicate gel around them. During alkaline activation, hydroxyl ions attack the Si–O–Si and Si–O–Al bonds in the aluminosilicate precursor, releasing soluble silicate and aluminate species that then reorganize and polycondense into an amorphous geopolymer gel [7,21]. However, the presence of intact spherical particles in Figure 12b,c shows that the 12 M–60 °C regime was insufficient for the complete dissolution of the FA particles. This incomplete reaction is also evident from the porous matrix and the weak particle–gel connection seen in the micrographs.

The gel regions indicated in Figure 12b–d can be interpreted as a reaction product of the sodium aluminosilicate hydrate type. In low-calcium Class F fly ash systems, the primary binding phase is usually N-A-S-H-type gel rather than calcium-rich C-A-S-H gel, because the low CaO content limits the formation of calcium-bearing reaction products [83]. The amorphous gel matrix shown in Figure 12c appears to connect the partially reacted particles, but open pores and unreacted spheres locally disrupt its continuity. This suggests that the reaction products were insufficient to fill the spaces between particles and the capillary voids.

Figure 12b,d show some void-like features within the FA-based geopolymer matrix. However, these should be interpreted carefully. Since SEM analyses were done on fractured surfaces, some rounded cavities might not be just open pores; they could also be imprints or pull-out cavities from unreacted or partially reacted spherical fly ash particles that detached during fracture or sample preparation. This is supported by the presence of intact and partially dissolved FA spheres, along with weak particle–gel interfaces seen in the same micrographs. As a result, these features are regarded as open pores and/or FA particle pull-out cavities, not solely true capillary pores. Prior research also indicates that curing temperature and activator concentration significantly affect reaction extent, matrix densification, and pore refinement in fly ash-based geopolymers [86].

However, their presence still suggests inadequate matrix continuity at the 12 M–60 °C activation condition. Whether these features are genuine open pores or cavities from particle pull-out, both indicate incomplete FA dissolution, weak bonds between residual particles and the aluminosilicate gel, and a limited formation of pore-filling reaction products. This interpretation aligns with the observed higher apparent porosity, increased capillary water absorption, and lower electrical resistivity in the FA–12M–60 °C mixture compared to more strongly activated FA mixtures.

**Figure 13:**
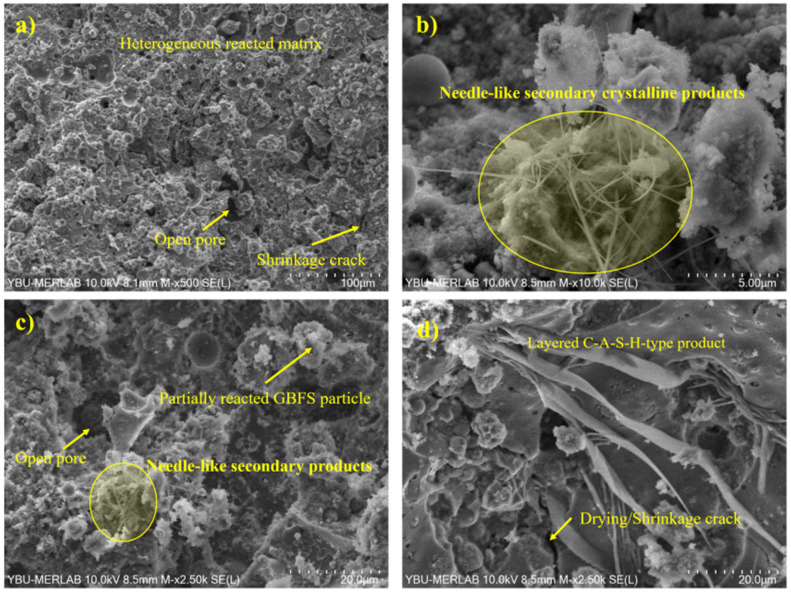
SEM images of the GBFS-based geopolymer mortar activated with 16 M NaOH and cured at 90 °C. (**a**) Heterogeneous reacted matrix with open pores and shrinkage cracks. (**b**) Needle-like secondary crystalline products. (**c**) Partially reacted GBFS particles with open pores. (**d**) Layered C-A-S-H-type products and drying/shrinkage cracks.

Figure 13 shows SEM images indicating that the GBFS-based geopolymer sample, activated with 16 M NaOH and cured at 90 °C, exhibits a heterogeneous internal structure. It features locally reacted gel regions, open pores, shrinkage cracks, and secondary crystalline formations, instead of a uniform, fully dense matrix. Because of GBFS’s high CaO content, the main binding products from alkali activation are likely Ca-rich gels similar to C-A-S-H or C-(N)-A-S-H phase [13,83]. The literature suggests that these reaction products may include low-Ca/Si ratio C-S-H/C-A-S-H gels, Na-containing C-(N)-A-S-H structures, and secondary hydrate phases [87]. Nevertheless, SEM images show that under 16 M–90 °C conditions, the formed reaction products do not create a continuous, dense gel network. Instead, they form a locally clustered structure with cracks intermittently disrupting the matrix.

Analyzing the overall matrix in Figure 13a reveals a heterogeneous reaction matrix, visible open pores, and cracks resulting from shrinkage. This indicates that, despite high alkalinity and elevated curing temperatures accelerating GBFS dissolution, the reaction does not occur in a controlled or uniform manner. In slag-based systems, a high calcium content can facilitate rapid formation of C-A-S-H and C-(N)-A-S-H phases early on. However, if the reaction accelerates too much, gel precipitates locally, moisture loss within the material accelerates, and microcracks may develop due to shrinkage [9,45]. Consequently, the open pores and cracks seen in Figure 13a align with the previously noted high capillary water absorption in this mixture, along with its lower mechanical strength compared to the 12 M–60 °C condition.

The needle-like structures in Figure 13b,c are likely secondary crystalline products. Nonetheless, the elevated NaOH molarity and increased local ion saturation at 90 °C curing may have led to the precipitation of secondary hydrate or Ca/Na-containing carbonation-related compounds. In alkali-activated slag systems, high alkali levels and carbonation propensity can promote the formation of hydrotalcite-like phases, carbonate products, or other secondary phases involving Ca, Al, and Mg, in addition to the primary gel. These needle-like formations might have compromised matrix homogeneity by forming localized crystallization zones rather than a dense, amorphous binder gel. Consequently, these areas suggest heterogeneous reaction products rather than a uniform gel network conducive to charge transfer. Figure 13c also shows partially reacted GBFS particles. This indicates that some slag grains did not fully dissolve despite the high molarity and high temperature. When rapid gel formation occurs on the surface of GBFS particles during alkali activation, the gel layer can partially restrict the alkali solution’s access to the particle’s inner regions. Thus, while reaction products form on the particle surface, the partially unreacted structure in the inner core can be preserved. This is consistent with the view stated in the literature that “the type and distribution of reaction products depend on the activator concentration, the glass phase of the slag and the curing conditions” [13,83]. Such partially reacted particles can form weak interfacial regions within the matrix, thereby limiting properties sensitive to crack propagation, especially flexural strength.

The layered C-A-S-H-type products and drying/shrinkage cracking observed in Figure 13d indicate that high-temperature curing has a detrimental effect on the microstructure of this sample. The layered, gel-like products suggest the formation of Ca-rich hydrate/gel phases due to GBFS’s high Ca content; however, cracks that interrupt these products weaken the matrix integrity. It has been reported that the high shrinkage tendency in alkali-activated slag systems may be associated with the development of a mesoporous structure, moisture loss, and the formation of a shrinkage-sensitive silicate gel [72]. Therefore, the drying shrinkage crack in Figure 13d provides significant microstructural evidence that the 16 M–90 °C condition is not optimal for the GBFS system. When evaluated alongside the study’s physical, mechanical, and transport results, these SEM findings reveal a significant cause-and-effect relationship. Although the best performance among GBFS-based samples was achieved under the 12 M–60 °C condition, the 16 M–90 °C condition yielded lower compressive strength and electrical resistivity and higher capillary water absorption. The open pores, shrinkage cracks, partially reacted slag particles, and needle-like secondary crystalline products observed in Figure 13 explain the microstructural basis of this performance loss. In other words, high molarity and high temperature accelerated the GBFS reaction; however, this acceleration led to local gel precipitation, moisture loss, and microcrack formation rather than homogeneous matrix condensation. Therefore, more aggressive activation conditions do not always yield better mechanical and durability performance in GBFS-binder systems; optimal performance can be achieved under more moderate curing and activation conditions that balance reaction rate and microstructural continuity.

**Figure 14:**
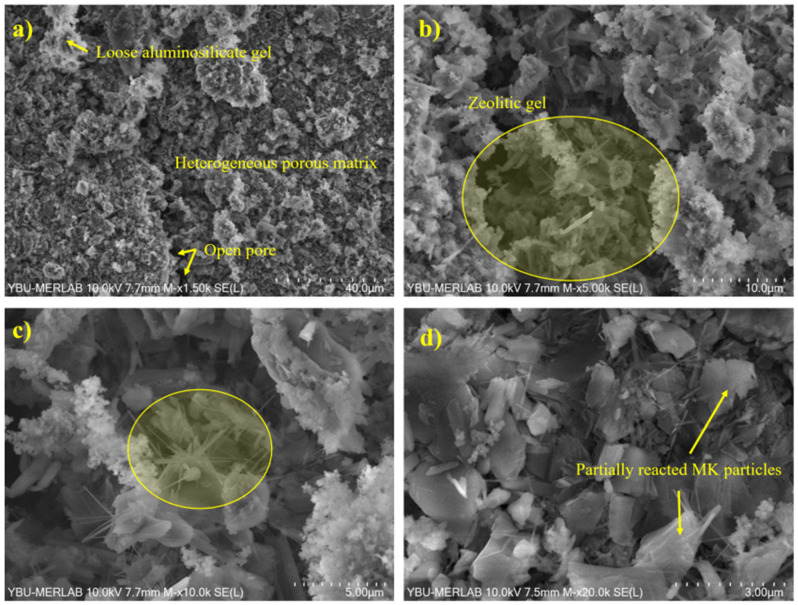
SEM images of the MK-based geopolymer mortar activated with 12 M NaOH and cured at 90 °C. (**a**) Heterogeneous porous matrix with loose aluminosilicate gel. (**b**) Zeolitic-type gel formation. (**c**) Needle-like zeolitic-type reaction products. (**d**) Partially reacted MK particles.

Figure 14’s SEM images reveal that the MK-based geopolymer sample, activated with 12 M NaOH and cured at 90 °C, did not form a dense, continuous aluminosilicate gel network. Instead, it shows a heterogeneous microstructure with loose gel clusters, open pores, zeolitic-like secondary products, and partially reacted metakaolin particles. Typically, the primary binder in metakaolin-based geopolymers is an amorphous N-A-S-H sodium-aluminosilicate gel. Still, its dense, continuous formation depends on factors such as the amount of alkali activator, solubility, Si/Al ratio, water content, and curing conditions [9,21]. The observed porous, loose structure suggests that the 12 M–90 °C setting does not fully realize the potential of the MK binder. Examination of the general microstructure in Figure 14a reveals a heterogeneous, porous matrix, open voids, and loose aluminosilicate gel formations. This structure indicates that the geopolymerization reaction, which begins with the dissolution of metakaolin particles, has occurred; however, the resulting gel products have not formed a continuous, compact skeleton throughout the matrix. In the literature, it has been reported that when sufficient activation is not achieved in metakaolin-based geopolymers, or when the activator concentration is insufficient for MK particles with high surface area, loose gel structures associated with incomplete geopolymerization, microvoids, and low electrical resistance can be observed [88]. Similarly, SEM-based studies have indicated that unreacted MK particles, microvoids, and honeycomb-like or incomplete gel products in metakaolin-containing geopolymer mortars can lead to the formation of a low-density matrix.

The needle-like and platy formations shown in Figure 14b,c can be considered zeolitic gel-like secondary crystalline products. These needle-like/platy products may represent locally formed sodium-aluminosilicate-based zeolitic-like phases or immature secondary reaction products in a highly alkaline environment. The literature indicates that the dissolution-rearrangement process of Si, Al, and Na species within the N-A-S-H gel structure can lead to zeolitic nucleation or the formation of crystalline products; temperature, reaction time, and alkalinity are key factors in developing these crystalline products [89]. Therefore, it is more appropriate to refer to these regions cautiously as “zeolitic-like secondary aluminosilicate products” rather than “zeolitic gel” in the article.

The visible, partially reacted MK particles in Figure 14d provide crucial evidence that the matrix is not fully polymerized. Metakaolin is typically viewed as a reactive precursor because of its high amorphous aluminosilicate content and large surface area; however, this reactivity also requires a significant amount of alkaline solution and water. When activation conditions are insufficient or unstable, an early gel layer can form on the surface of MK particles, potentially hindering further dissolution by blocking alkaline access to the internal regions [48]. However, under more aggressive activation conditions, the GBFS-based mixtures showed higher apparent porosity, higher water absorption, lower strength, and higher capillary water absorption than the 12 M–60 °C mixture. These results indicate a less favorable pore structure under 16 M and/or 90 °C conditions. Although drying shrinkage was not directly measured in this study, the SEM observations of the GBFS–16M–90 °C mixture revealed crack-like features, open pores, and a heterogeneous reaction matrix. Therefore, the performance reduction under high molarity and high-temperature curing is attributed to rapid reaction, non-uniform gel formation, possible moisture-loss-related microcracking, and increased pore connectivity, rather than solely to measured shrinkage. This explains why the current sample did not develop such a structure despite curing at 90 °C. These SEM results strongly confirm the study’s previous findings from the physical, mechanical, and transport analyses. The MK–12M–90 °C sample’s high apparent porosity, water absorption, capillary water absorption, low electrical resistivity, and elevated chloride-ion penetrability directly relate to its open pores, discontinuous gel network, partially reacted MK particles, and zeolitic-like secondary products shown in Figure 14. Its low compressive and flexural strength also stems from an underdeveloped load-bearing N-A-S-H gel network. Essentially, the 12 M–90 °C condition partially facilitated MK dissolution, but the reaction products failed to form a uniform, dense binder phase in the matrix.

The improvements seen in MK-based mixtures with increasing NaOH molarity from 12 M to 16M should be considered alongside the physical, mechanical, and transport property results. SEM/EDS analysis of the MK–12M–90 °C mixture revealed a heterogeneous matrix with loose aluminosilicate gel areas, partially reacted MK particles, open pores, and zeolitic-like secondary products, indicating that 12 M activation was insufficient to form a dense, continuous N-A-S-H-type gel network. Although SEM/EDS was not conducted on the MK–16M–90 °C mixture, its significantly lower porosity and water absorption, higher oven-dry density, improved compressive and flexural strengths, reduced capillary water absorption, increased electrical resistivity, and lower charge transfer collectively suggest more effective matrix densification at 16 M–90 °C. Thus, the improved geopolymerization in MK-16 M mixtures is interpreted through microstructural evidence supporting performance, rather than direct phase confirmation from SEM.

**Figure 15:**
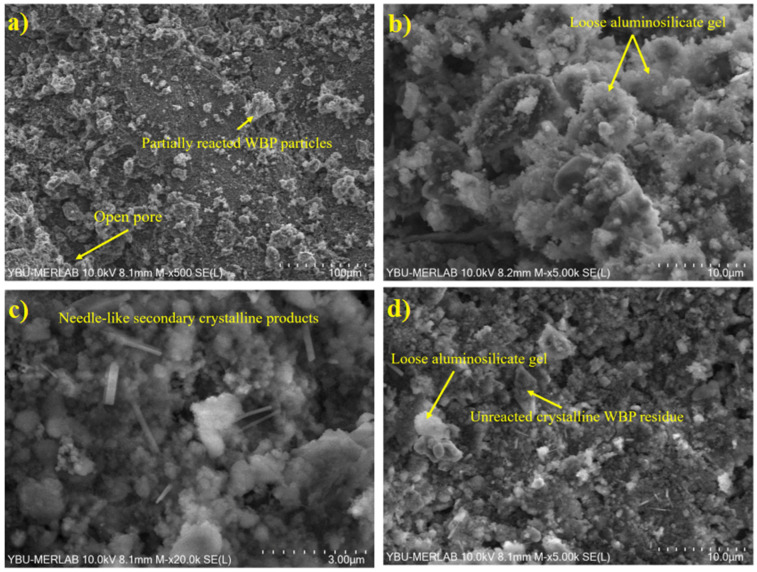
SEM images of the WBP-based geopolymer mortar activated with 16 M NaOH and cured at 60 °C. (**a**) Partially reacted WBP particles and open pores. (**b**) Loose aluminosilicate gel formation. (**c**) Needle-like secondary crystalline products. (**d**) Loose aluminosilicate gel with unreacted crystalline WBP residue.

The SEM images in Figure 15 show that the WBP-based geopolymer sample, activated with 16 M NaOH and cured at 60 °C, exhibits a highly heterogeneous, loosely aggregated gel, open pores, and an incompletely condensed microstructure. Although waste brick dust is a precursor rich in SiO_2_ and Al_2_O_3_, it generally contains quartz, mullite, hematite, and other crystalline phases due to its fired-clay origin. Therefore, the solubility of WBP in an alkaline environment may be more limited than that of more amorphous precursors such as FA or MK [35,49]. The partially reacted WBP particles, unreacted crystalline residues, loose aluminosilicate gel, and open pores observed in Figure 15 clearly demonstrate the microstructural response to this limited reactivity. At low magnification in Figure 15a, the overall matrix structure appears disordered, with partially reacted WBP particles and open pores, rather than a homogeneous, compact binder phase. This suggests that 16 M NaOH initiated surface dissolution of WBP. Still, the curing temperature of 60 °C was insufficient to dissolve the crystalline/coarse WBP particles fully and form a continuous gel network. The literature reports that the mechanical performance of WBP-based alkali-activated systems largely depends on the amorphous phase content of WBP, its fineness, activator concentration, and curing conditions; and that low reactive/crystalline residue limit matrix condensation [72]. Therefore, the open pores observed in Figure 15a are consistent with the high capillary water absorption and low electrical resistivity tendency of this sample.

The loose aluminosilicate gel clumps shown in Figure 15b indicate that WBP undergoes alkali activation; however, the resulting reaction products fail to form a dense, continuous network. Such loose gel clumps can be observed in systems where sufficient Si–Al species are not dissolved, or where the dissolved species cannot repolymerize homogeneously. The angular, crystalline particle structure of WBP may have limited the effective filling of interparticle spaces by the reaction products. This is one of the main microstructural reasons why WBP, despite its lower cost, does not perform as well as FA, GBFS, or optimal MK systems in mechanical and transport performance. The needle-like secondary crystalline products shown in Figure 15c suggest that local ion saturation increases due to the high NaOH molarity, allowing some secondary products to precipitate within the matrix. Therefore, it is safer to describe these structures as “needle-like secondary crystalline products” in the article. These secondary products can reduce matrix homogeneity by forming localized crystallization regions rather than acting as a compact binder gel. It has been reported that inadequate or unstable reaction conditions in WBP-based systems can result in porous, heterogeneous microstructures by limiting gel formation [71].

In Figure 15d, visible remains of unreacted crystalline WBP are observed alongside the loose aluminosilicate gel. These remnants indicate that the more stable, less reactive components of WBP remain undissolved in the alkaline environment. Phases such as quartz and mullite have limited solubility during alkaline activation, which can impede WBP’s transformation into a fully developed binder phase [49,72]. As a result, these areas can create weak interfacial zones and charge transfer discontinuities within the matrix. This microstructural characteristic likely contributes to the lower compressive and flexural strengths and the higher permeability observed in the WBP–16M–60 °C sample. Figure 15 indicates that 16 M NaOH partially promotes the alkaline activation of WBP, but curing at 60 °C is insufficient to develop a dense, continuous geopolymer matrix. The presence of loose gel clusters, open pores, unreacted crystalline WBP residues, and needle-like secondary products in the sample underscores WBP’s limitations, given its low reactivity and high crystallinity. These observations align with earlier physical and mechanical results: the WBP–16M–60 °C sample exhibited relatively high porosity and capillary water absorption, low electrical resistivity, and limited strength. Consequently, the primary microstructural factors limiting performance are incomplete WBP dissolution, discontinuous gel formation, and the resulting open-pore structure.

#### 3.5.2. EDS Examinations

In this study, EDS was not used as a phase-identification technique alone. EDS spectra were interpreted alongside SEM morphology, precursor chemistry, and the expected reaction products of alkali-activated linker systems. In this context, Na–Al–Si-rich regions were interpreted as N-A-S-H-type gel-like reaction products, while Ca–Si–Al-rich regions were associated with C-A-S-H/C-(N)-A-S-H-type reaction products.

**Figure 16:**
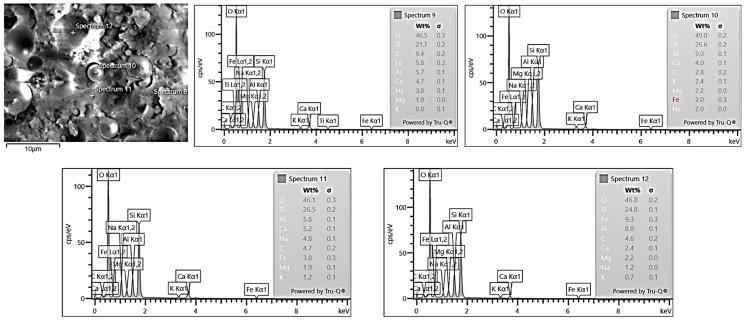
EDS analysis of the FA-based geopolymer mortar activated with 12 M NaOH and cured at 60 °C.

Figure 16 shows that an aluminosilicate gel based on Si–Al–Na formed in the FA sample activated with 12 M NaOH and cured at 60 °C, although the matrix remained chemically heterogeneous. The detection of Al and Na, along with high O and Si levels in all spectra, indicates partial dissolution of FA in the alkaline environment and the formation of an N-A-S-H-type gel [21]. However, notable differences between the spectra suggest that the reaction products were not evenly distributed, with some FA particles partially reacted and mineral residues unreacted. Spectrum 10 displays aluminosilicate gel with Si (26.6%), Al (9.0%), and Na (2.0%), while Spectrum 12’s high Fe content (9.3%) suggests this point may be an Fe-rich unreacted or partially reacted FA residue. The Ca detected in Spectra 9 and 11 points to the presence of hybrid gel or raw ash-derived Ca phases in the low-calcium FA system. These findings align with SEM images showing unreacted FA spheres, open pores, and a discontinuous gel structure. Thus, FA geopolymerization commenced at 12 M–60 °C, but limited strength and increased water/ion transport imply that full matrix densification was not achieved [44,77].

**Figure 17:**
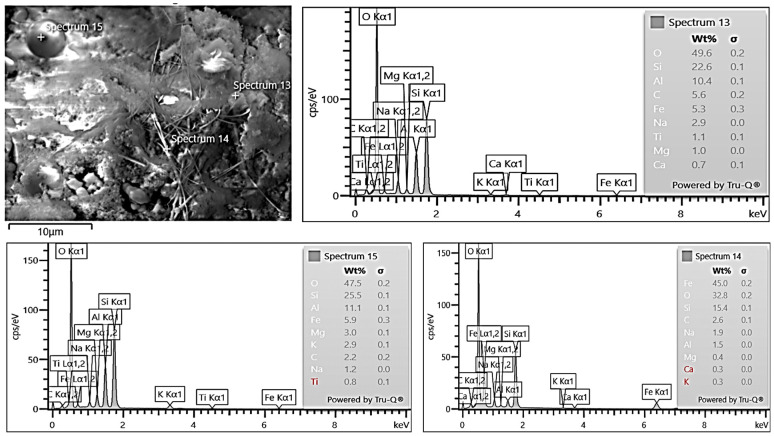
EDS analysis of the GBFS-based geopolymer mortar activated with 16 M NaOH and cured at 90 °C.

EDS analyses shown in Figure 17 indicate that the GBFS-based sample, activated with 16 M NaOH and cured at 90 °C, develops a chemically heterogeneous reaction matrix. The limited Na content, together with high O, Si, and Al in Spectra 13 and 15, indicates the formation of aluminosilicate-based reaction products. However, the relatively low Ca content in these two spectra suggests that the classical Ca-rich C-A-S-H gel is not dominant in the analyzed regions; instead, low-Ca Si–Al-rich hybrid/aluminosilicate gel domains have developed [13,77]. In Spectrum 14, the Fe content of 45.0% indicates that this point is either a Fe-rich secondary crystalline product or a Fe-rich slag residue rather than the main binder gel phase. These findings are consistent with the needle-like secondary products, heterogeneous reaction matrix, and shrinkage cracks observed in the SEM images. Therefore, high molarity and 90 °C curing accelerated the reaction of GBFS; however, rather than forming a homogeneous and compact C-A-S-H/C-(N)-A-S-H matrix, it produced low-Ca aluminosilicate gel regions, Fe-rich local products, and a fractured/heterogeneous microstructure. This explains the microchemical basis for the GBFS–16M–90 °C sample showing more limited mechanical and transport performance compared to the 12 M–60 °C condition [90,91].

**Figure 18:**
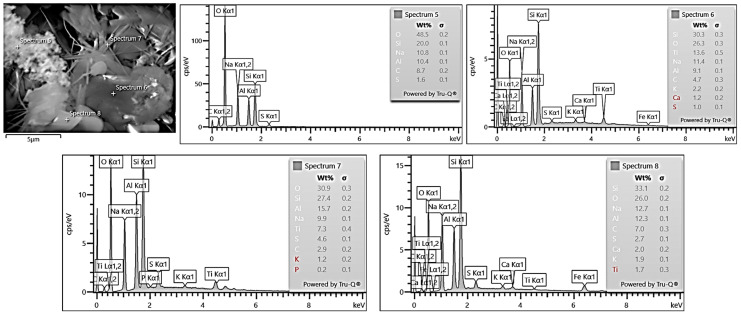
EDS analysis of the MK-based geopolymer mortar activated with 12 M NaOH and cured at 90 °C.

The EDS analyses in Figure 18 reveal that the MK-based sample, activated with 12 M NaOH and cured at 90 °C, forms Na–Al–Si-rich reaction products. Spectrum 5, Spectrum 7, and Spectrum 8 show high levels of Si, Al, and Na, indicating partial dissolution of MK in the alkaline environment and the start of N-A-S-H aluminosilicate gel formation [21]. The notable chemical differences between spectra and the Si/Al ratio variation from about 1.68 to 3.20 suggest that the reaction products are heterogeneously distributed. The elevated Si and Na in Spectrum 8 may be linked to zeolitic-like secondary aluminosilicate products seen in SEM [48]. The high Ti content (13.6%) detected in Spectrum 6 indicates that this area corresponds to a Ti-rich residue or secondary products, rather than the main binder gel phase. This microchemical heterogeneity aligns with the loose gel structure observed in SEM images, partially reacted MK particles, and open pores. Hence, MK geopolymerization began under 12 M–90 °C conditions, but the sample’s mechanical and transport properties remained limited because a dense, continuous N-A-S-H gel network did not form.

**Figure 19:**
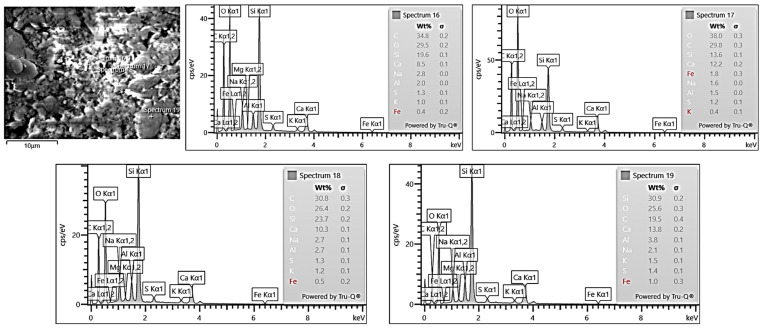
EDS analysis of the WBP-based geopolymer mortar activated with 16 M NaOH and cured at 60 °C.

The EDS analyses shown in Figure 19 indicate that the WBP-based sample activated with 16 M NaOH and cured at 60 °C is mainly composed of regions rich in Si–Ca with limited Al–Na content. Spectra 16–19 reveal Si levels between 13.6–30.9%, Ca levels from 8.5–13.8%, while Al and Na are present at 1.5–3.8% and 1.6–2.8%, respectively. This composition suggests the presence of partially reacted WBP particles, Si–Ca-rich residues, and a scarcity of Ca/Na-containing aluminosilicate reaction products, rather than a fully developed, homogeneous N-A-S-H gel network [35,49]. The high Si/Al ratios imply that silica-rich crystalline phases and poorly reacted WBP residues remain embedded within the matrix. A high C signal may be associated with partial carbonation of calcium-rich areas, hydrocarbon contamination picked up on the surface during sample preparation, and/or a small contribution from the conductive carbon strip used to stabilize the sample. This chemical heterogeneity aligns with the observed loose gel clusters, open pores, and unreacted crystalline WBP residues seen in SEM images. Consequently, while the 16 M–60 °C activation condition initiated alkaline activation of WBP, it was insufficient for creating a dense, continuous bonding matrix, contributing to the sample’s limited mechanical strength and high transport tendencies [18].

From an architectural-material perspective, the differences observed between precursor systems may also influence their suitability for specific building applications. For example, MK- and FA-based mixtures exhibiting lower porosity and higher durability may be advantageous for exterior façade components and prefabricated building elements exposed to environmental actions. In contrast, WBP-based systems, despite their relatively lower mechanical performance, still offer potential for non-structural architectural products where waste valorization and environmental sustainability are prioritized, such as partition blocks, paving materials, or low-load interior applications.

## 4. Conclusions

This study carefully examined how precursor type, NaOH molarity, and thermal curing temperature influence the physical, mechanical, transport, microstructural, and cost-performance characteristics of geopolymer mortars made from FA, GBFS, MK, and WBP. The findings showed that a single activation rule cannot describe the performance of geopolymer mixes. Instead, each precursor responds uniquely based on its chemical makeup, amorphous phase content, particle size, and its sensitivity to alkalinity and curing temperature. The key conclusions are summarized as follows:

The optimal activation condition heavily depended on the precursor. FA and MK performed better with increased NaOH molarity and higher curing temperatures, while GBFS reached its peak performance under the milder 12 M–60 °C condition. WBP experienced some improvement at 16 M–90 °C, but its performance was still constrained by its coarser particle size and more crystalline structure.

MK activated with 16 M NaOH and cured at 90 °C showed the best overall performance. This mixture achieved the lowest apparent porosity (6.1%), water absorption (5.4%), and the highest oven-dry density (2194 kg/m^3^), 28-day compressive strength (25.8 MPa), and flexural strength (3.43 MPa). These results indicate that sufficient alkalinity and thermal activation enabled the formation of a dense, continuous aluminosilicate matrix in the MK system.

Transport-related properties exhibited similar performance patterns. The MK–16M–90 °C mixture demonstrated the lowest capillary water absorption at 1.88 kg/m^2^, the highest electrical resistivity at 248.00 kΩ·cm, and the lowest charge passed according to ASTM C1202 [27] with 177 C. The FA–16M–90 °C mixture also showed good resistance to transport processes, whereas WBP-based mixtures exhibited higher capillary absorption and lower resistivity, likely due to incomplete matrix densification.

Correlation analysis confirmed that the pore structure largely determines the main engineering properties. Apparent porosity was strongly inversely related to 28-day compressive strength. Moreover, mixtures with higher strength typically showed lower charge passed and greater electrical resistivity, suggesting better matrix densification and fewer ion-transport pathways. These findings indicate that densifying the matrix and reducing connected porosity are crucial factors influencing both mechanical strength and transport properties.

The cost analysis showed that the lowest material cost did not necessarily correspond to the best cost-performance efficiency. Although WBP-based mixtures were the lowest-cost to produce, their low strength led to poor cost index values. The MK–16M–90 °C mixture offered the best cost index at $4.1 per MPa, with FA–16M–90 °C and GBFS–12M–60 °C following as technically and economically viable options.

Overall, the results indicate that a universal activation regime cannot be applied to all geopolymer precursors. Instead, NaOH molarity and curing temperature should be optimized based on precursor characteristics, amorphous content, particle size, and calcium content. Among the tested systems, MK–16M–90 °C was the most efficient in terms of combined mechanical, transport, microstructural, and cost-performance behavior.

## Figures and Tables

**Table 1:** The chemical compositions of binders.

Binder	CaO	SiO_2_	Al_2_O_3_	Fe_2_O_3_	MgO	Na_2_O	K_2_O	SO_3_	LOI ^1^
FA	4.3	56.3	26.4	7.9	1.1	0.4	0.7	0.6	2.2
GBFS	42.3	32.2	10.3	0.6	10.8	0.3	0.9	0.2	1.3
MK	0.3	51.9	43.4	1.7	0.3	0.4	0.9	0.2	0.8
WBP	11.8	48.9	24.2	7.2	4.1	0.6	1.4	1.1	0.6

^1^ Loss on ignition.

**Table 2:** Mixing parameters and material quantities (kg/m^3^).

Binder Type	NaOH Molarity	Binder	NaOH	Na_2_SiO_3_	Extra Water	Quartz Sand
**Fly ash**	12 M	600	120	300	50	932
**Blast furnace slag**		600	120	300	50	1092
**Metakaolin**		600	120	300	50	994
**Waste brick powder**		600	120	300	50	1003
**Fly ash**	16 M	600	120	300	70	883
**Blast furnace slag**		600	120	300	70	1035
**Metakaolin**		600	120	300	70	942
**Waste brick powder**		600	120	300	70	988

**Table 3:** Activator and liquid-to-binder ratios used in the mixture design.

NaOH Molarity	Na_2_SiO_3_/NaOH	Extra Water/Binder	Activator/Binder	Total Liquid/Binder
12 M	2.50	0.083	0.700	0.783
16 M	2.50	0.117	0.700	0.817

**Table 4:** Nominal and dilution-adjusted NaOH concentrations used in the mixtures.

NaOH Stock Solution	NaOH Solution Dosage (kg/m^3^)	Measured Solution Density (kg/L)	NaOH Solution Volume (L/m^3^)	Extra Water (kg/m^3^)	Extra Water Volume (L/m^3^)	Dilution-Adjusted NaOH Concentration (M)
12 M	120	1.38	86.96	50	50	7.62
16 M	120	1.43	83.92	70	70	8.72

**Table 5:** Evaluation of the electrical resistivity and chlorine permeability performance of geopolymer samples.

Binder Type	Molarity-Curing	Electrical Resistivity (kΩ·cm)	Risk of Corrosion	Charge Passed (C)	Chloride-Ion Penetrability Class
FA	12 M–60 °C	45.67	Negligible	611	Very low
FA	12 M–90 °C	76.54	Negligible	276	Very low
FA	16 M–60 °C	72.93	Negligible	302	Very low
FA	16 M–90 °C	182.67	Negligible	232	Very low
GBFS	12 M–60 °C	98.67	Negligible	376	Very low
GBFS	12 M–90 °C	55.65	Negligible	366	Very low
GBFS	16 M–60 °C	56.75	Negligible	359	Very low
GBFS	16 M–90 °C	52.35	Negligible	389	Very low
MK	12 M–60 °C	13.58	Low	1499	Low
MK	12 M–90 °C	13.53	Low	1527	Low
MK	16 M–60 °C	112.46	Negligible	254	Very low
MK	16 M–90 °C	248.00	Negligible	177	Very low
WBP	12 M–60 °C	13.07	Low	1632	Low
WBP	12 M–90 °C	29.46	Negligible	1021	Low
WBP	16 M–60 °C	13.44	Low	1515	Low
WBP	16 M–90 °C	41.52	Negligible	673	Very low

**Table 6:** Unit prices ($/ton) of the materials used in the preparation of mixtures.

NaOH (12 M)	NaOH (16 M)	Na_2_SiO_3_	Quartz Sand	GBFS	FA	MK	WBP	Water
120	129	132	19	45	31	39	11	0.74

## Data Availability

The original contributions presented in this study are included in the article. Further inquiries can be directed to the corresponding author.
